# Curcumin Prevents Degeneration of Medial Septal Cholinergic Neurons Produced by Parkinsonism-Inducing Neurotoxin 1-Methyl-4-Phenylpyridinium

**DOI:** 10.3390/molecules31213814

**Published:** 2026-10-22

**Authors:** Paola Lenzi, Michela Ferrucci, Gloria Lazzeri, Alessandra Polzella, Marco Scotto, Francesca Biagioni, Paola Binda, Carla L. Busceti, Francesco Fornai

**Affiliations:** 1 Department of Translational Research and New Technologies in Medicine and Surgery, University of Pisa, 56126 Pisa, Italy; paola.lenzi@unipi.it (P.L.); michela.ferrucci@unipi.it (M.F.); gloria.lazzeri@unipi.it (G.L.); paola.binda@unipi.it (P.B.); 2 School of Psychology, Sigmund Freud Privat Universität Wien, Milan Campus, 20143 Milan, Italy; 62103058@mail.sfu.ac.at; 3 Department Translational Medicine, Sant’Anna School of Advanced Studies, 56126 Pisa, Italy; marco.scotto@santannapisa.it; 4 Istituto di Ricovero e Cura a Carattere Scientifico (IRCCS) Neuromed, 86077 Pozzilli, Italy; francesca.biagioni@neuromed.it (F.B.); carla.busceti@neuromed.it (C.L.B.)

**Keywords:** Parkinson’s disease, medial septal neurons, mitochondria, ACh neurons, PINK1, parkin, LC3-II, phytochemicals, neuroprotection, neurorescue

## Abstract

The neurotoxin 1-methyl-4-phenyl-1,2,3,6-tetrahydropyridine is converted into the active metabolite 1-methyl-4-phenylpyridinium (MPP^+^), which damages dopamine (DA)-containing neurons of Substantia Nigra, and norepinephrine (NE)-containing neurons of Locus Coeruleus, thus mimicking the pathology of Parkinson’s disease (PD). In PD, cognitive impairment often occurs, which may be due to a loss of acetylcholine (ACh)-containing neurons within the basal forebrain. In turn, this may result from either direct damage to ACh neurons or indirect damage through loss of their DA and/or NE innervation. In the present study, we used neurons from the medial septal nucleus (MSN), which grow in the absence of DA or NE innervation, to assess the direct effects of MPP^+^ on MSN-derived ACh neurons. Administration of MPP^+^ directly damages MSN-derived neurons, as evidenced by neuronal loss and dramatic mitochondrial alterations. The phytochemical curcumin administered before MPP^+^ prevents ACh neuronal damage and mitochondrial derangement. Curcumin administration following MPP^+^ still promotes neuronal survival while reshaping both damaged mitochondria and cellular processes of ACh neurons. These latter effects are consistent with the expression of key proteins such as PINK1, Parkin, and LC3-II and activation of autophagy flux.

## 1. Introduction

The neurotoxin 1-methyl-4-phenyl-1,2,3,6-tetrahydropyridine (MPTP) was identified as a parkinsonism-inducing substance in humans in the late 1970s and early 1980s [1,2,3,4,5,6,7]. Administering MPTP to various animal species, including rodents [8,9,10,11] and primates [12,13,14,15], mimics most features of Parkinson’s disease (PD) [2,3]. These effects are due to its active metabolite 1-methyl-4-phenylpyridinium (MPP^+^), which is selectively taken up by dopamine (DA) and norepinephrine (NE) neurons [16,17,18]. Once inside the neuron, MPP+ causes severe mitochondrial damage [19,20,21,22,23]. This produces the loss of DA-containing neurons in the Substantia Nigra (SN), impairing movement control [24,25]. In PD patients, cognitive decline is also common, as reported by several studies of the past two decades [26,27,28,29,30,31,32,33,34]. This might be due to a loss of DA innervation in the cognitive channel of the basal ganglia [35,36,37,38,39,40,41,42]. Alternatively, a loss of NE innervation from the locus coeruleus (LC) may contribute to cognitive decline [43]. Still in PD patients, damage to cholinergic (ACh) neurons, which affects cognition, often occurs [44,45]. It is debated whether such damage to ACh neurons may be a consequence of the loss of NE-LC neurons [46] or mesolimbic DA systems [47], or it may be directly produced by MPP^+^ on vulnerable medial septal nucleus (MSN) ACh neurons projecting in the septo-hippocampal pathway. Some findings report that MPTP causes a loss of ACh neurons in the proencephalon [48,49,50,51]. However, in vivo approaches leave open whether this results from damage to DA/NE neurons or whether MPTP may directly destroy MSN ACh neurons through its active metabolite MPP^+^. To answer this question, in the present study we used neurons from MSN deprived of DA or NE innervation [52,53]. Here, the direct effects of MPP^+^ on ACh neurons were tested. Since the first part of the study indicates that MPP^+^ does produce direct damage to MSN, in the second part of this research work, administration of the phytochemical curcumin before or after MPP^+^ was tested as a neuroprotective strategy. Curcumin is a phytochemical with known protective effects in neurodegenerative disorders [54,55,56,57,58,59]. This occurs through several mechanisms [60,61,62,63,64] involving antioxidant effects, mitophagy, and mitochondrial biogenesis [65,66,67,68,69,70]. Thus, the novelty of the present study extends to the potential disease-modifying effects of curcumin to counteract MSN Ach damage [71,72,73]. In detail, curcumin was administered either before (to test neuroprotection) or after (to test potential neurorescue/disease-modifying effects) MPP^+^ administration. Concomitantly, curcumin, which is a safe compound [74,75], was assessed as a potential strategy to preserve subcellular morphology, induce plastic events such as cell branching, and promote mitochondrial rejuvenation. In detail, curcumin was assessed concerning promoting/preserving/rescuing cell phenotype and cell clearance.

## 2. Results

### 2.1. Dose-Dependent Neurotoxicity of MPP^+^ for MSN Neurons

**Figure 1:**
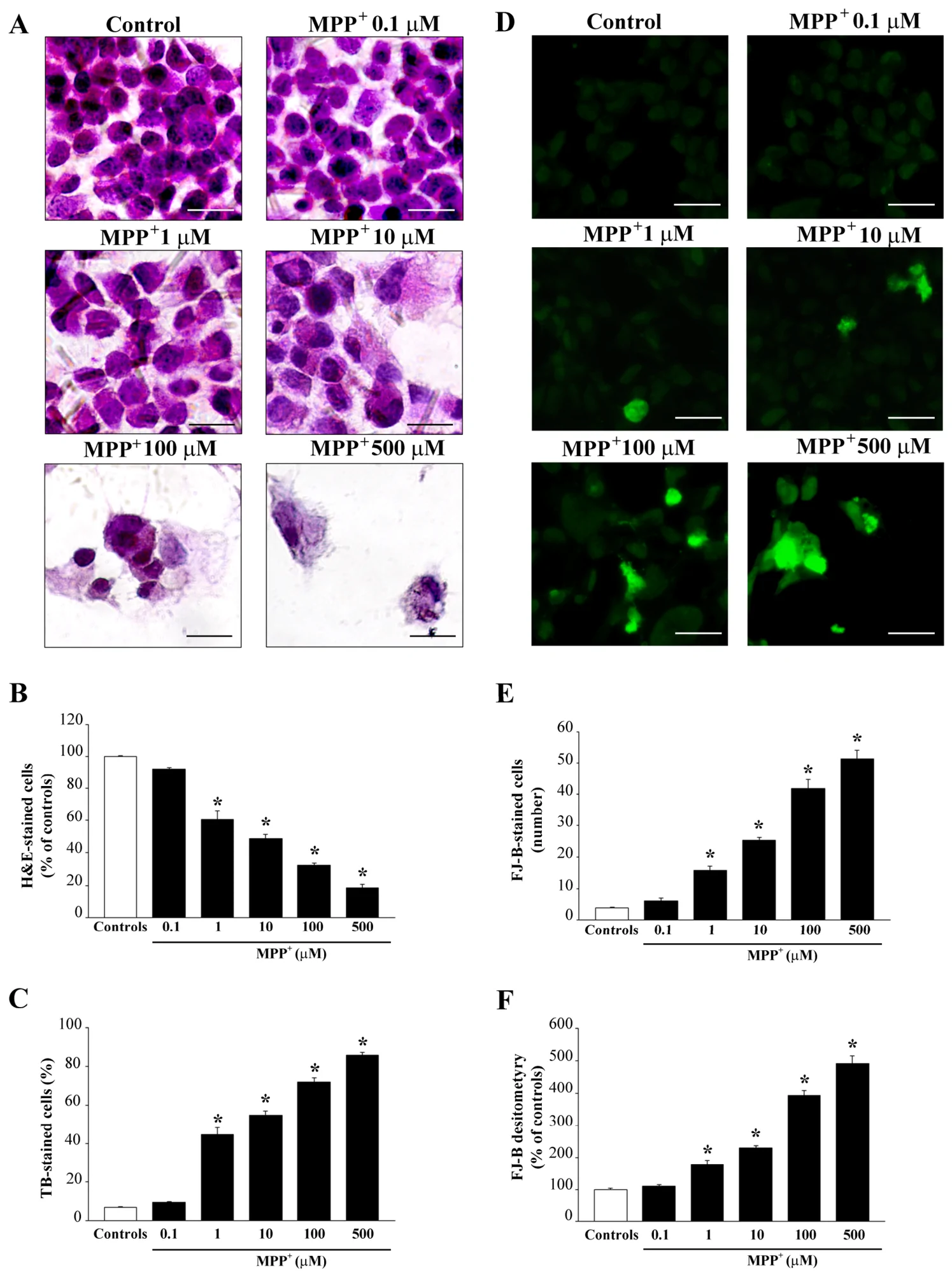
MPP^+^ dose-dependently reduces neuron viability. (**A**) Representative pictures from H&E show control SN56 neurons. MPP^+^-treated neurons are altered starting from the dose of 1 μM MPP^+^, increasing up to 500 μM MPP^+^. (**B**) The graph reports the dose-dependent decrease in H&E-stained neurons. Data are expressed as the mean percentage ± S.E.M. of controls (considering controls = 100%). Graph (**C**) reports the percentage of TB-stained cells. Data are the mean percentage ± S.E.M. of TB-stained cells. (**D**) Representative pictures of cells stained with the fluorescent dye FJ-B, which stains degenerating neurons. The graphs in (**E**,**F**) report the count of FJ-B fluorescent cells and the mean FJ-B fluorescence intensity per cell, respectively. Densitometry of FJ-B-positive cells was reported as the mean percentage ± S.E.M. * *p* < 0.05 compared with controls. Scale bars = (**A**) 22 μm; (**D**) 28 μm.

SN56 cell culture represents a neuronal population that ubiquitously expresses typical ACh antigens in each cell, such as the enzyme choline acetyltransferase (ChAT, Supplementary Figure S1). Figure 1A shows representative pictures from H&E-stained SN56 ACh neurons. Control cells form a continuous layer and appear round or polygonal in shape, with large, intensely hematoxylin-stained nuclei and strongly eosinophilic cytosol. After treatment with increasing doses of MPP^+^ (0.1 μM up to 500 μM), the cell layer loses continuity, and single scattered neurons appear larger in size, irregular in shape, with a pale cytosol. These changes are evident starting from 1 μM MPP^+^ and increase dose-dependently (Figure 1A). The count of H&E-stained cells is shown in the graph in Figure 1B, which reports that MPP^+^ reduces the number of SN56 cells. Cell loss occurs significantly, starting at 1 μM MPP^+^, and increases up to 500 μM MPP^+^ (graph in Figure 1B). To assess MPP^+^-induced cell degeneration, we used the marker for dying cells, Trypan Blue (TB). This dye penetrates dying cells through the discontinuous cell membrane (see Section 4). The graph in Figure 1C reports a dose-dependent increase in TB-stained cells, which indicates that dying neurons increase with increasing MPP^+^ (Figure 1C). TB-positive neurons are evident even at 1 μM MPP^+^ (Figure 1C). A similar trend is evident when SN56 cells are stained using the greenish fluorescent dye FluoroJade-B (FJ-B), which stains degenerating neurons. Representative pictures in Figure 1D show that FJ-B fluorescence, due to polyamines [76,77,78], becomes increasingly intense as the dose of MPP^+^ rises to 500 μM (Figure 1D). The graphs in Figure 1E,F report the count of FJ-B fluorescent cells and the mean percentage ± S.E.M. of FJ-B fluorescence intensity per cell, respectively. These data confirm that MPP^+^ administration causes cell degeneration dose-dependently, starting at the dose of 1 μM. This is remarkable when considering that the vulnerability of these ACh neurons to MPP^+^ is ten-fold the vulnerability of catecholamine cells (Supplementary Figure S2).

Apart from slight discrepancies, the amount of cell damage produced by MPP^+^ is comparable across different techniques.

### 2.2. Dose-Dependent Mitochondrial Damage Produced by MPP^+^ (Mitotracker Green, MTR-G = Total Mitochondria; Mitotracker Red, MTR-R = Healthy Mitochondria; Transmission Electron Microscopy, TEM)

**Figure 2:**
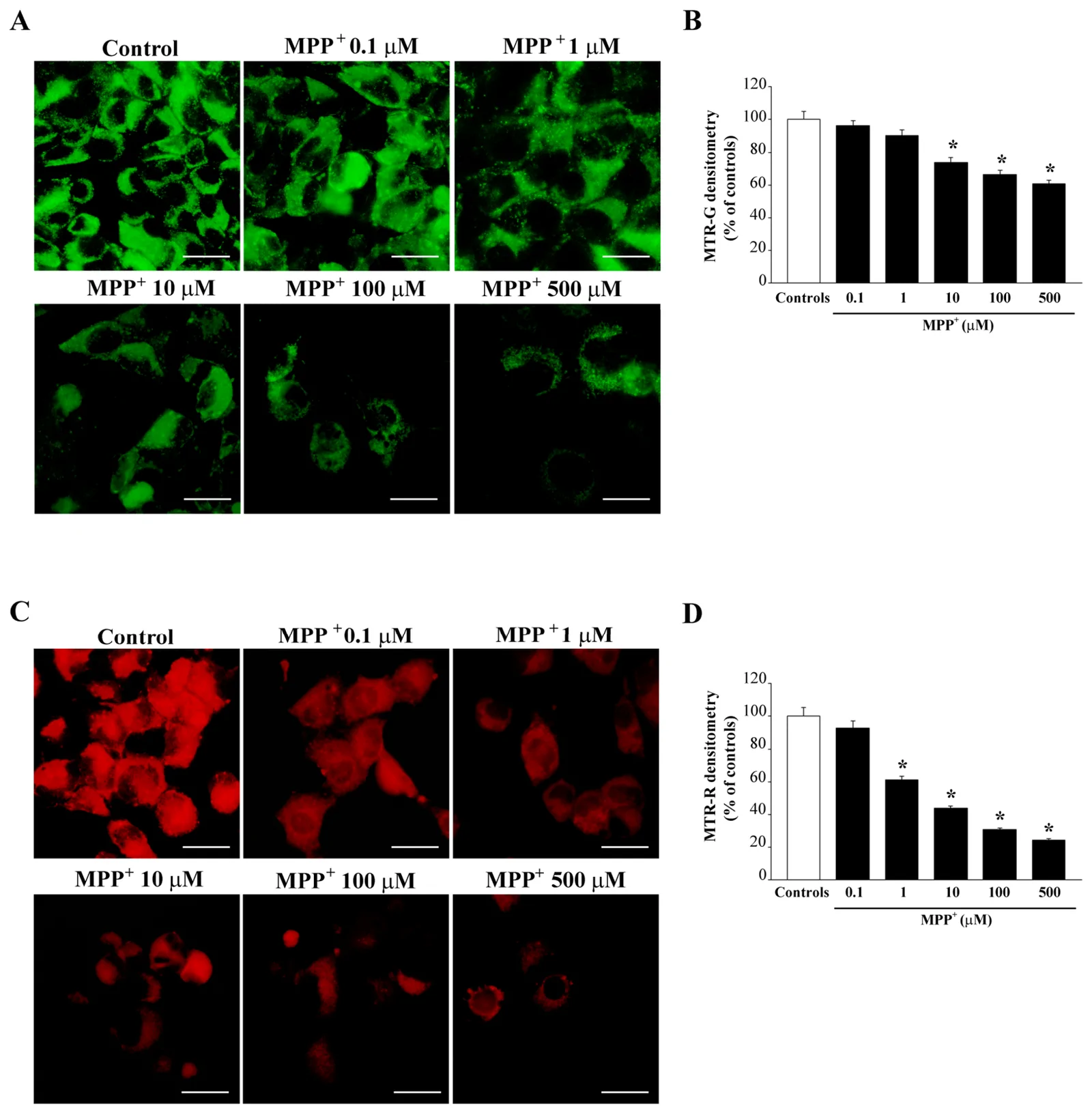
MPP^+^ dose-dependently alters mitochondria (MTR-G = total and MTR-R = healthy). (**A**) Representative pictures showing a dose-dependent decrease in MTR-G fluorescence. The graph in (**B**) reports the MPP^+^-induced decrease in fluorescence intensity. (**C**) Representative pictures from cells stained with MTR-R showing a dose-dependent decrease in healthy mitochondria. The graph in (**D**) reports the count of MTR-R fluorescence intensity, which is produced by MPP^+^ starting from a dose of 1 μM. Data are expressed as the mean percentage ± S.E.M. of densitometry. * *p* < 0.05 compared with controls. Scale bars = (**A**,**C**) 14 μm.

MTR-G staining reveals a decrease in the number of total mitochondria, which is significant starting at a dose of 10 μM MPP^+^, as shown in representative Figure 2A and quantified in the graph in Figure 2B. Since MTR-G does not differentiate between healthy and damaged mitochondria [79,80,81], the results suggest that MPP^+^ decreases all mitochondria. To identify specifically healthy mitochondria, MTR-R staining was carried out [79,80,81]. MTR-R staining indicates that MPP^+^ produces a decrease in healthy mitochondria, as shown in representative pictures in Figure 2C and the graph in Figure 2D. A decrease in healthy mitochondria starts at the dose of 1 μM MPP^+^ and progresses up to 500 μM (Figure 2D).

**Figure 3:**
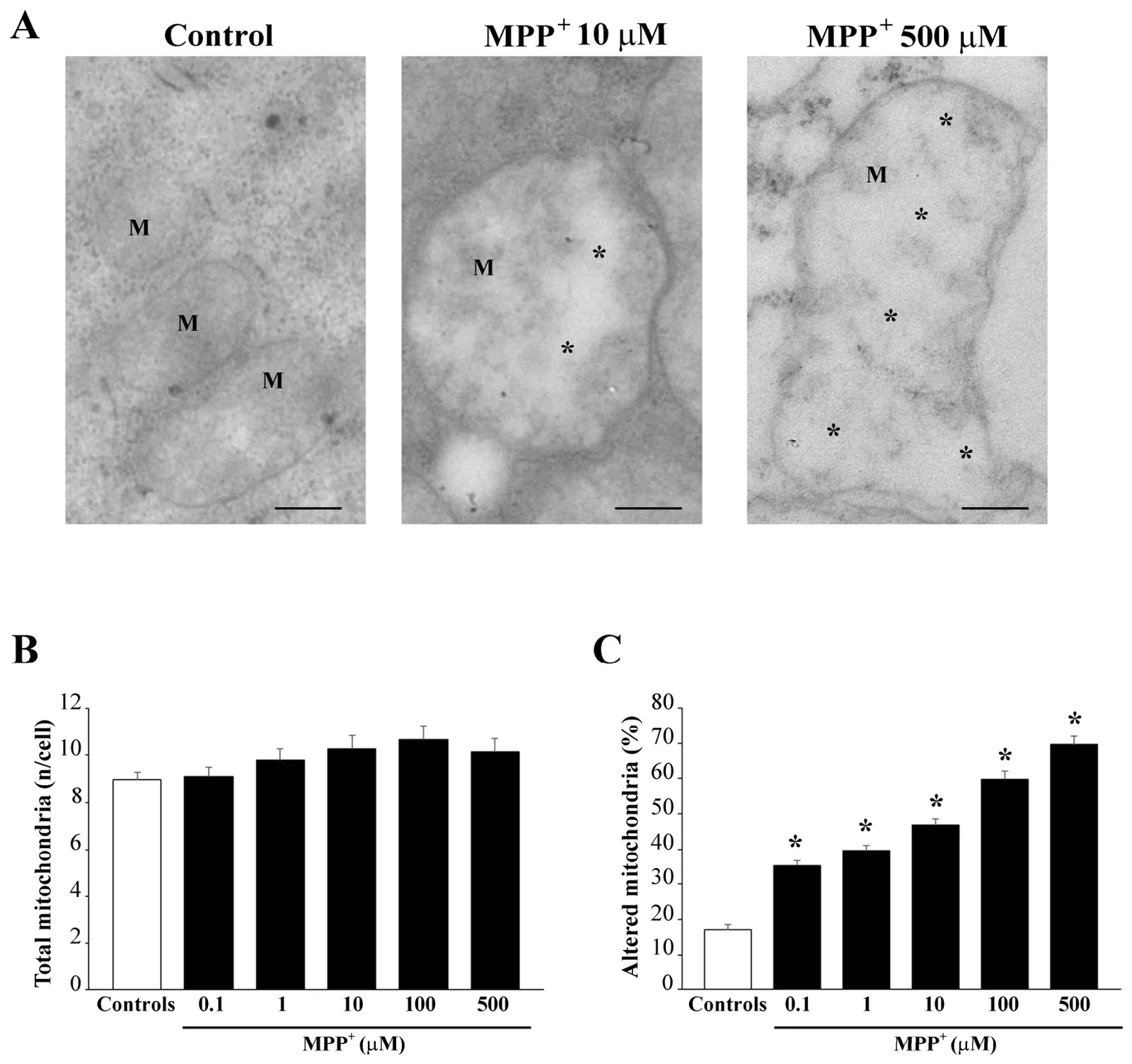
MPP^+^ dose-dependently produces mitochondrial damage at TEM. (**A**) Representative micrographs from a control neuron and from 10 μM and 500 μM MPP^+^-treated neurons showing dose-dependent mitochondrial alterations, which feature diluted matrix and broken cristae (*). The graph in (**B**) reports the total number of mitochondria, which remains steady as the dose of MPP^+^ increases up to 500 μM. In contrast, the graph in (**C**), which reports the percentage of altered mitochondria, indicates the occurrence of a dose-dependent increase in mitochondrial damage. This is significant starting from the dose of 0.1 μM. Data are expressed as the mean ± S.E.M. of mitochondria per cell or the mean percentage ± S.E.M. of altered mitochondria per cell. M = mitochondria. * *p* < 0.05 compared with controls. Scale bars = 140 nm.

The decrease in total mitochondria visualized at MTR-G contrasts with a steady mitochondrial number detected at TEM (representative micrographs of Figure 3A and graphs of Figure 3B,C). The explanation for such a discrepancy is provided by ultrastructural analysis indicating an increase in damaged mitochondria (Figure 3). In detail, a *continuum* of damage severity ranging from slight mitochondrial damage to severe mitochondrial alterations depending on MPP^+^ doses is counted at TEM (Figure 3A and graph of Figure 3C). This provides an explanation for the decreased ability to retain MTR-G despite the total number of mitochondria counted at TEM being steady. The decrease in total mitochondria, as revealed by MTR-G staining, occurs at higher MPP^+^ concentrations (starting from 10 μM) compared with the dose needed to reduce healthy mitochondria (1 μM MPP^+^). This is consistent with the fact that only severe mitochondrial damage impairs mitochondrial retention of MTR-G. Ultrastructural data confirm a severe decrease in healthy mitochondria, which is already significant at the dose of 0.1 μM (Figure 3C).

In detail, the optical fluorescence density of MTR-G and MTR-R is influenced by multiple factors, and it may not directly correlate with mitochondrial number. This is why we further specified mitochondrial areas, as an index of mitochondrial mass, by TEM measurements. One needs to keep in mind that data from MTR-G and MTR-R do not represent quantitative data, and they do not allow inference about the actual mitochondrial number/mass. Instead, MTR-G and MTR-R represent mere semi-quantitative evidence suggesting a change in mitochondrial mass (total = green; healthy = red). This is specifically addressed by adding TEM analysis based on ultrastructural morphometry, where mitochondrial area in each cell, which is directly related to mitochondrial volume and mass, indicates a severe decrease in healthy mitochondrial mass following MPP^+^, which is in line with MTR-R optical data. In detail, TEM analysis shows that despite preserving the total mitochondrial number, MPP^+^ produces a few giant dysfunctional mitochondria (supposedly corresponding to organelles affected by MPP^+^) within a vast majority of small (likely newly synthesized, likely compensatory) mitochondria. Thus, ultrastructural morphometry indicates that the decrease in healthy mitochondrial mass was way more pronounced compared with the decrease in total mitochondrial mass/area (which fits very well with semi-quantitative data provided by MTR-R compared with MTR-G). In fact, the giant swollen mitochondria were excluded from the count of healthy mitochondrial mass.

These dose-dependent effects of MPP^+^ on the percentage of mitochondrial alterations assessed at TEM were similar to the effects on cell degeneration observed with H&E, TB and FJ-B staining (Figure 1A–F).

These data were further analysed in the presence of curcumin.

### 2.3. Effects of Curcumin on Cell Viability

To select the specific dose of curcumin to be used to evaluate neuroprotection against MPP^+^-induced toxicity, a dose–response curve was carried out by treating MSN neurons with increasing amounts of curcumin, ranging from 0.1 to 50 μM, for 72 h (Supplementary Figure S3). As shown by representative H&E-stained cells and the graph reporting the percentage of TB-positive cells, curcumin doses ranging from 0.1 to 10 μM are not toxic for SN56 cells, while toxic effects are produced only when curcumin is used at the dose of 50 μM (Supplementary Figure S3). Therefore, the dose of 10 μM curcumin was selected for experiments aimed at counteracting MPP^+^ neurotoxicity. Control cells were treated with vehicle (the culture medium containing 0.1% or 0.5% DMSO).

The safety of the culture medium containing up to 0.5% DMSO, where the highest dose of curcumin was dissolved (50 μM), was assessed, and the results are shown in Supplementary Figure S4. These data show that treatment of SN56 cells with DMSO up to 0.5% does not affect cell survival compared with control cells.

**Figure 4:**
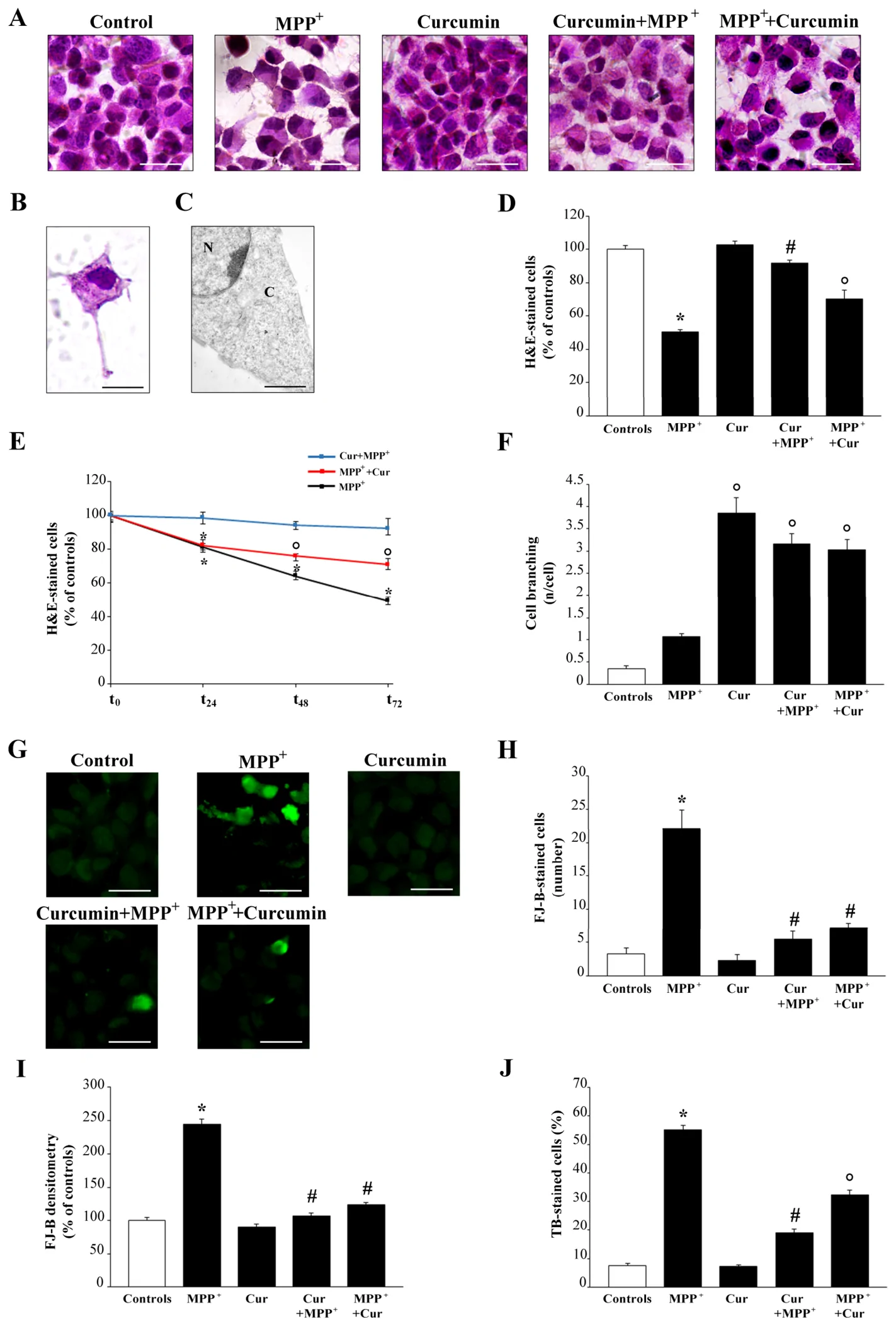
Curcumin counteracts MPP^+^-induced neurodegeneration. (**A**) Representative pictures from H&E-stained neurons, following various treatments. MPP^+^ was administered at a dose of 10 μM; curcumin (10 μM) was administered alone or in combined treatments, either before (curcumin + MPP^+^) or after (MPP^+^ + curcumin) MPP^+^. When curcumin was administered, neurons produced cell branching as shown at higher magnification in (**B**) or at TEM in (**C**). The graph in (**D**) reports the protective effects of curcumin against MPP^+^-induced toxicity. The graph in (**E**) reports time-dependent neurodegeneration following MPP^+^ either alone or in combination with curcumin. Graph (**F**) reports the counts of cell branching. (**G**) Representative pictures of FJ-B fluorescence and graphs shown in (**H**,**I**) report the effects of curcumin either before or after MPP^+^. The graph in (**J**) reports the same groups stained with TB. C = cytosol; Cur = curcumin; N = nucleus. * *p* < 0.05 compared with controls; ^#^ *p* < 0.05 compared with MPP^+^; ° *p* < 0.05 compared with controls and MPP^+^. Scale bars: (**A**) 29 μm; (**B**) 11 μm; (**C**) 50 nm; (**G**) 21 μm.

When MSN neurons are administered curcumin at a dose of 10 μM, this produced neuroprotection against 10 μM MPP^+^ (Figure 4). Such protection is complete, as shown in representative pictures of Figure 4A. At higher magnification, when curcumin was administered, dense cell branching is evident (Figure 4B,C). These plastic effects are replicated in curcumin + MPP^+^-treated cells. To appreciate these fine morphological events, the cell cultures were analysed by TEM (Figure 4C), where curcumin produces cell branching (processes) featuring dense endoplasmic reticulum (Figure 4C).

The graph in Figure 4D reports the count of H&E-stained cells administered curcumin, either before or after MPP^+^, showing that curcumin significantly reduces the loss of SN56 cells induced by MPP^+^. In detail, cell viability in cells treated with curcumin either before or after MPP^+^ was 91.75% ± 1.67% or 70.19% ± 5.39% of controls, respectively, while MPP^+^ alone reduced viable cells to 50.26% ± 1.49% of controls (graph in Figure 4D).

When curcumin was administered at 24 h following MPP^+^, a significant rescue of cell viability was documented. The addition of curcumin at 24 h after MPP^+^ reduced the development of full damage and brought the amount of MPP+ toxicity back to roughly half. In detail, the damage produced by MPP^+^ at 48 h was attenuated from 34% to 24% when curcumin was added, and from 50% to 30% at 72 h (Figure 4E). Remarkably, neuronal recovery induced by curcumin was accompanied by marked cell branching (Figure 4F). In detail, in curcumin-treated cells, the mean number of cell processes per cell was 3.8 ± 0.3 compared with 0.3 ± 0.1 in controls. This was similar when curcumin was administered either before or after MPP^+^ (3.2 ± 0.2 and 3.0 ± 0.2, respectively) (Figure 4F). When we measured the length of the longest cell process in cells from each experimental group, we found that cells treated with curcumin alone possessed the longest cell process. An increased length of cell branching was also observed when curcumin was administered either before or after MPP^+^ (Table 1).

Protection and rescue of cell viability produced by curcumin were replicated when cell cultures were stained with FJ-B (representative pictures of Figure 4G and graphs of Figure 4H,I) and TB (graph of Figure 4J).

### 2.4. Effects of Curcumin and MPP^+^ on Mitochondria

**Figure 5:**
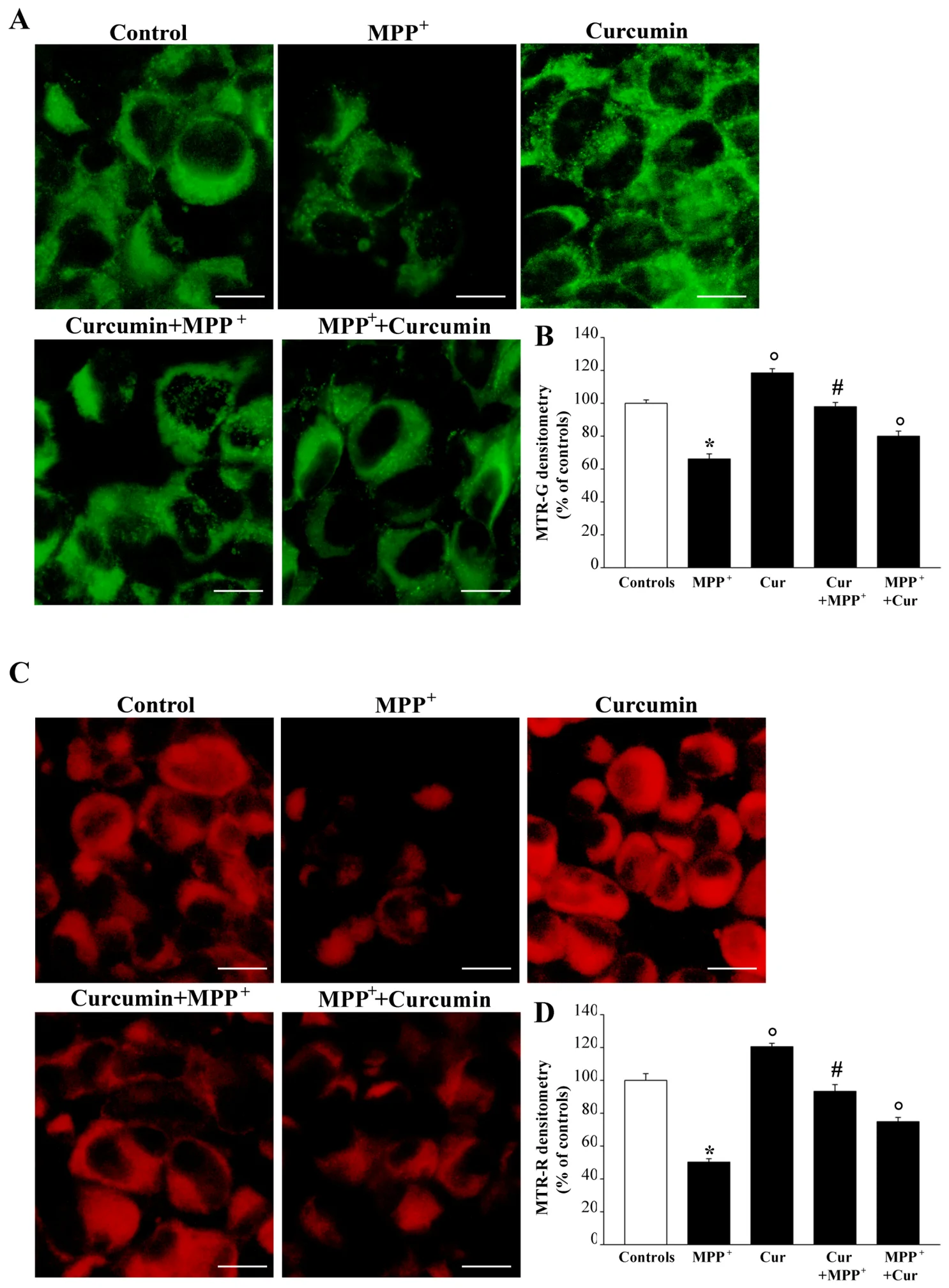
Curcumin counteracts MPP^+^-induced mitochondrial damage (MTR-G = total and MTR-R = healthy). (**A**) Representative pictures of MTR-G fluorescent cells, which are counted in the graph in (**B**), indicate curcumin-induced protection or rescue. (**C**) Representative pictures of the same groups following MTR-R. Curcumin counteracts the decrease in healthy mitochondria as reported in the graph in (**D**). Data are the mean percentage ± S.E.M. Cur = curcumin. * *p* < 0.05 compared with controls; ^#^ *p* < 0.05 compared with MPP^+^; ° *p* < 0.05 compared with controls and MPP^+^. Scale bars = (**A**,**C**) 8 μm.

When MPP^+^ was administered at high doses, MTR-G fluorescence was markedly reduced. In detail, a slight decrease was noticed already for MPP^+^ compared with controls at the low doses of 0.1 μM and 1 μM, which was significant for MPP^+^ at the dose of 10 μM up to 500 μM. This was evident in representative pictures of Figure 2A, and it is reported in the graph of Figure 2B. The dose of MPP^+^ 10 μM was selected to be challenged with curcumin for mitochondrial status. MPP^+^ 10 μM markedly decreases total MTR-G stained mitochondrial mass (Figure 5A). This was fully prevented when curcumin was administered before MPP^+^. A significant recovery of mitochondrial mass was observed with curcumin, even when it was administered at 24 h following MPP^+^ (representative pictures and graph in Figure 5A and Figure 5B, respectively). Mitochondrial preservation by curcumin was evident by staining the cells with MTR-R, which selectively marks healthy mitochondria. In detail, curcumin alone increased the fluorescence produced by healthy mitochondria compared with controls (representative pictures and graph in Figure 5C and Figure 5D, respectively). Similarly, curcumin administered before MPP^+^ fully prevents the loss of healthy mitochondria, and it partially rescues/mitigate mitochondrial damage progression when administered 24 h after MPP^+^ (representative pictures and graph in Figure 5C and Figure 5D, respectively).

**Figure 6:**
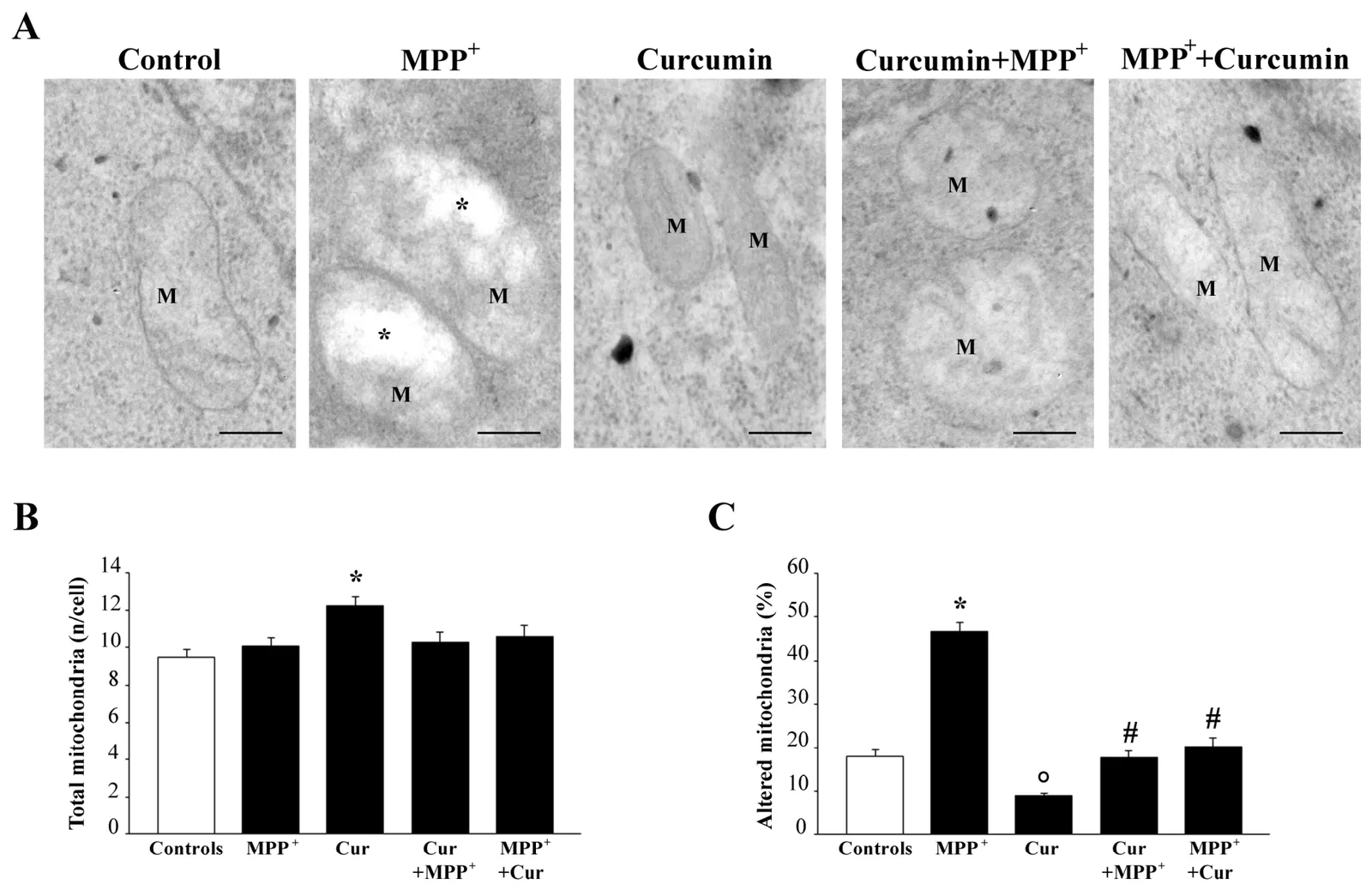
Curcumin prevents MPP^+^-induced mitochondrial alterations. (**A**) Representative TEM micrographs following various treatments. The graphs in (**B**,**C**) report the total mitochondria and the percentage of altered mitochondria (*), respectively. Data are expressed as the mean ± S.E.M. of mitochondria per cell or the mean percentage ± S.E.M. of altered mitochondria per cell. Cur = curcumin; M = mitochondria. * *p* < 0.05 compared with controls; ^#^ *p* < 0.05 compared with MPP^+^; ° *p* < 0.05 compared with controls and MPP^+^. Scale bars = 140 nm.

These data, obtained by qualitative measurement based on optical density, were confirmed by counting the total number of mitochondria and by assessing mitochondrial damage through plain ultrastructural morphometry (representative pictures in Figure 6A and graphs in Figure 6B,C). MPP^+^ does not decrease the mitochondrial number. Rather, the number of mitochondria remains steady (graph of Figure 6B), though an increased amount of mitochondrial alterations is evident (graph of Figure 6C), which leads to an alteration in the total mitochondrial area/mass. The protective effects of curcumin, when characterized by TEM, show that mitochondrial alterations were greatly prevented, resulting in a healthy mitochondrial area/mass (representative pictures of Figure 6A and graphs of Figure 6B,C). The effects of curcumin are still significant when curcumin is administered 24 h after MPP^+^ (Figure 6A and graphs of Figure 6B,C). Adding curcumin always elevates healthy mitochondrial area/mass and total mitochondrial mass, which is still compatible with mitochondrial histochemistry with MTR-G and MTR-R. Since the mitochondrial number is not modified, while the mitochondrial area is altered following MPP^+^ or curcumin, this leads to a decrease in mitochondrial mass following MPP^+^, which is conversely increased by curcumin (which increases the number of healthy and total mitochondria and the healthy and total mitochondrial area/mass, Supplementary Figure S5). These gross data are in line with data obtained following staining with MTR-G and MTR-R. Slight discrepancies exist between light and electron microscopy, which are likely to be sustained by additional issues such as membrane potential and staining procedures, or the nature of the probes.

In detail, mitochondrial shape is improved by curcumin. Matrix dilution, which is severe following MPP^+^, is significantly reduced by either pre- or post-treatment with curcumin. Curcumin alone has beneficial effects on mitochondrial structure even in baseline conditions. Similar effects are observed concerning vacuolization. In fact, curcumin reduces fragmented cristae, promotes the close placement of the inner and outer mitochondrial membranes, abolishes laminated membranes, and counteracts mitochondrial swelling, which is fostered by MPP^+^ (representative pictures and graph of Figure 6).

**Figure 7:**
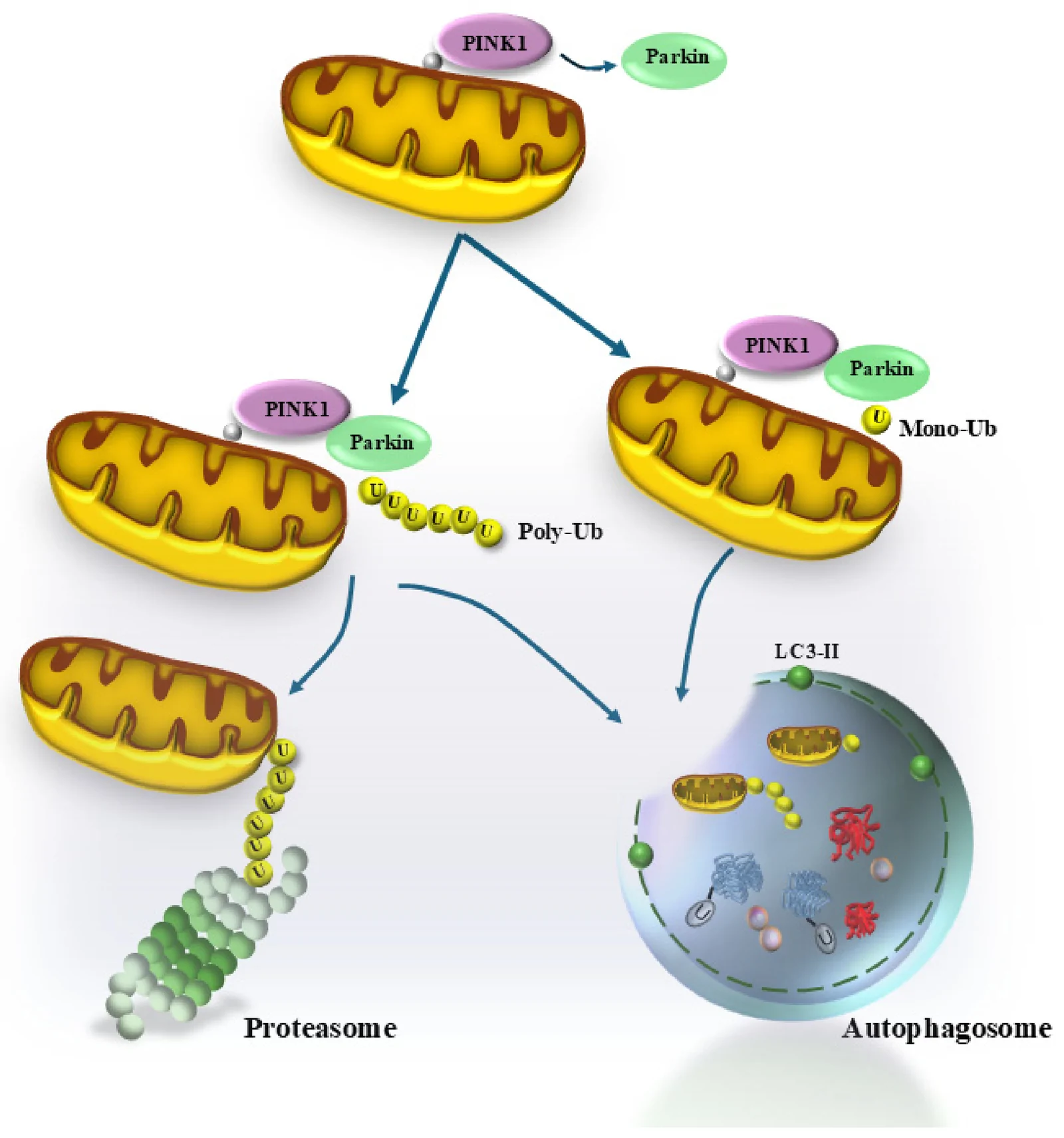
Cartoon showing the role of PINK1 and Parkin in mitochondrial removal. The occurrence of damaged mitochondria is sensed by the expression of PINK1 on the outer mitochondrial membrane (and sometimes elsewhere in the mitochondria). The expression of PINK1 serves as a powerful signal to recruit Parkin, which acts as a ubiquitin-ligase. This is essential to form mono- or poly-ubiquitin chains binding altered mitochondria. Mono-ubiquitinated mitochondria are delivered to LC3-II-positive autophagic vacuoles or nascent autophagosomes, while poly-ubiquitin chains may either enter the autophagy pathway or bind the proteasome (P19S subunit) for proteasomal processing. LC3-II = lipidated microtubule-associated protein 1A/1B-light chain 3; Mono-Ub = mono-ubiquitin; Poly-Ub = poly-ubiquitin.

In order to better decipher the deleterious effects produced by MPP^+^ and the beneficial action of curcumin on the fate of mitochondria, we stained two seminal proteins regulating mitochondrial turnover, such as PINK1 and Parkin. PINK1 is a powerful sensor of mitochondrial damage [82,83,84], and its presence on the outer mitochondrial membrane is associated with severe damage that needs repair through mitochondrial removal. The presence of mitochondrial PINK1 is sensed by a fundamental protein such as Parkin, which binds PINK1 and anchors mitochondria to mono-ubiquitin or poly-ubiquitin chains to promote their degradation (cartoon of Figure 7).

**Figure 8:**
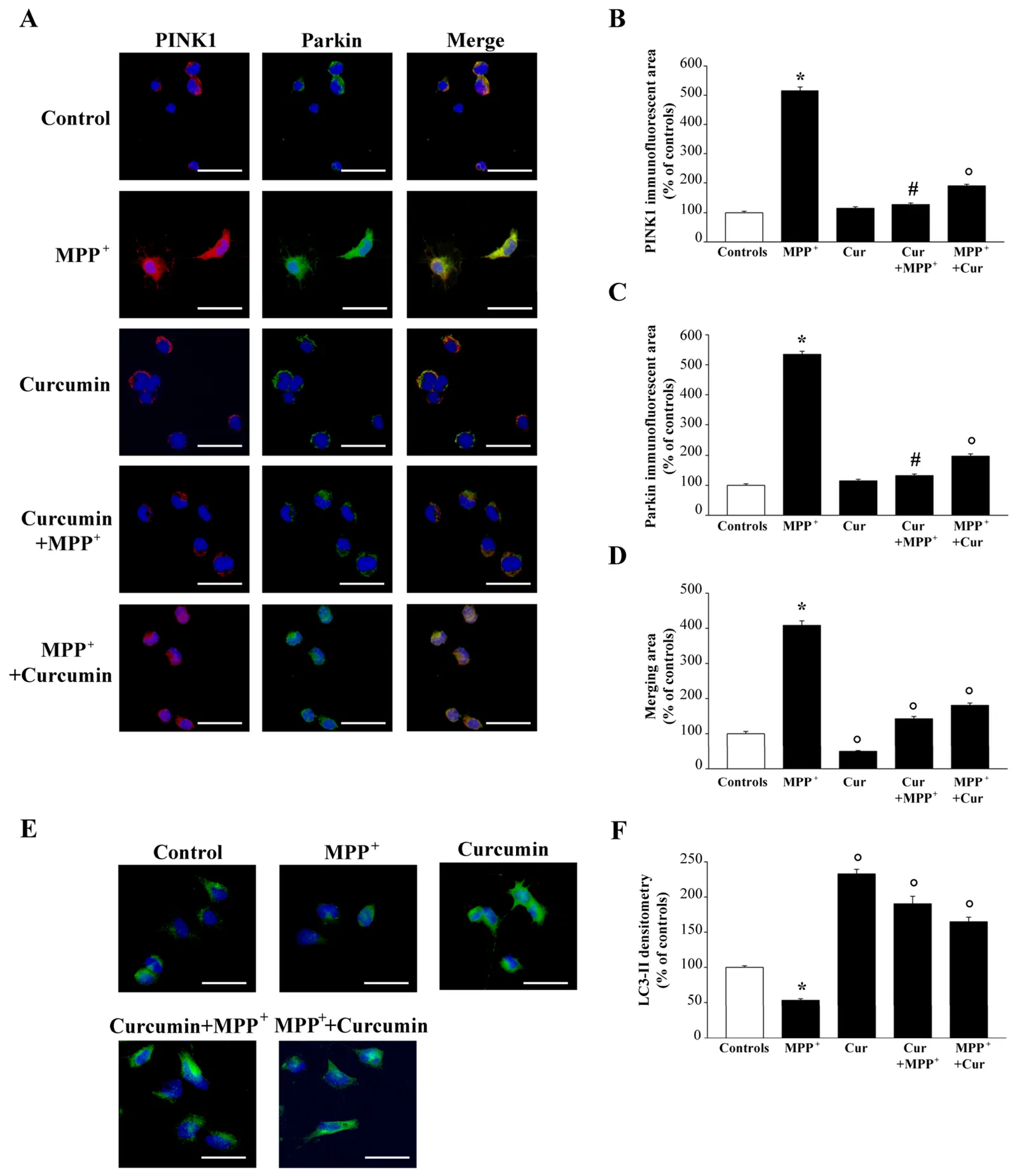
MPP^+^ increases PINK1 and Parkin expression, while it decreases LC3-II. The effects of MPP^+^ on PINK1, Parkin, and LC3-II are suppressed by curcumin. This is evident from representative pictures in (**A**) showing immunostaining for PINK1, Parkin, and their merging. Graphs in (**B**–**D**) report immunofluorescent areas for PINK1, Parkin, and their merging in each group, respectively. (**E**) Representative pictures from each group show LC3-II immunofluorescence, which is quantified in the graph in (**F**). Cur = curcumin. * *p* < 0.05 compared with controls; # *p* < 0.05 compared with MPP^+^; ° *p* < 0.05 compared with controls and MPP^+^. Scale bars = (**A**) 42 μm; (**E**) 32 μm.

The effects of MPP^+^ are remarkable in increasing the expression of PINK1 and Parkin, along with their merging (representative pictures and graphs of Figure 8A–D). This is attenuated by curcumin, either when administered before or after MPP^+^. These findings are likely to be the consequence of massive protection by curcumin against mitochondrial damage, which prevents mitochondrial recruitment of either PINK1 or Parkin.

Since the fate of mitochondria is bound to the shuttling of these organelles towards nascent autophagy structures, we stained the lipidated isoform of microtubule-associated protein 1A/1B-light chain 3 (LC3-II, Figure 8E,F). In line with a marked stimulation of cell clearance induced by curcumin as an autophagy inducer [62,65,73,85,86,87,88,89,90,91,92,93,94,95,96,97,98,99,100,101,102,103,104], the cells administered curcumin feature an excess of LC3-II immunostaining. This is in excess even compared with controls, while curcumin counteracts the suppression of LC3-II immunostaining produced by MPP^+^ (representative pictures and graphs of Figure 8E,F) [105].

Altogether, data on mitochondrial status along with PINK1, Parkin, and LC3-II immunostaining provide evidence for severe deleterious effects of MPP^+^ on mitochondrial structure, which is sensed by PINK1 and communicated to Parkin to initiate mitochondrial removal. However, a failure of cell clearance produced by MPP^+^ is evident. This is consistent with a decrease in lipidated LC3-II, suggesting that MPP^+^ impairs autophagy flux (Supplementary Figure S6). This may limit the progression towards beneficial mitochondrial clearance. These effects of MPP^+^ are counteracted by curcumin. This is in line with the stoichiometry counts of immunogold particles for PINK1, Parkin (Supplementary Figure S7), and LC3-II (Supplementary Figure S8), which provide quantitative protein levels in situ.

One may hypothesize that, in MPP^+^-treated cells, an excess of PINK1/Parkin-dependent ubiquitinated substrates (such as altered mitochondria) occurs, while these structures need to be rejuvenated. Curcumin is expected to provide such a beneficial effect.

## 3. Discussion

We found that MPP^+^ produces dose-dependent cell death in SN56 MSN cells, which starts at a dose of 1 μM. This is quite surprising since it indicates that the sensitivity of ACh neurons to the toxic effects of MPP^+^ is almost ten-fold higher compared with catecholamine cells (Supplementary Figure S2). The higher sensitivity of ACh neurons compared with catecholamine neurons to parkinsonism-inducing neurotoxins relies on similar subcellular targets. In detail, within spared cells, mitochondrial ultrastructure is markedly affected. Mitochondrial alterations are evident at sub-lethal doses of MPP^+^, and they are detailed according to specific morphometry.

The administration of curcumin was carried out to test a phytochemical potentially useful to improve the progression of ACh cell degeneration. In detail, curcumin at 10 μM prevents MPP^+^-induced degeneration of ACh neurons. Such a dose of curcumin promotes recovery of ACh neurons when administered at 24 h following MPP^+^. These effects are grounded, at least in part, on the beneficial role of curcumin on mitochondrial pathology. In detail, despite acting as an ACh neurotoxin, MPP^+^ exerts its molecular mechanisms similarly in ACh and DA neurons by targeting mitochondria. These effects are prevented and significantly reversed by curcumin. The mitochondrial toxicity exerted by MPP^+^ on ACh neurons is not related to a defect in the PINK1/Parkin pathway, which is strongly recruited here. Rather, it is likely to depend on a block within downstream steps in cell clearing systems. This hypothesis is consistent with a decreased amount of the active protein LC3-II [106] and a decreased autophagy flux measured in the present study. Thus, the MSN ACh neurons undergo direct damage following MPP^+^, which occurs independently of concomitant effects on DA and NE systems, which are ruled out in the present experimental conditions. ACh damage is prevented and/or rescued by direct effects of curcumin on MSN ACh neurons. This is consistent with the protective effects of curcumin in neurodegeneration, including AD and PD [107,108,109]. Similarly, curcumin administration may halt cognitive decline and memory impairment during aging [110,111].

Since in vivo MSN ACh neurons project to the hippocampus and related limbic circuitry, the present findings suggest that cognitive alterations in PD are likely to be sustained by specific damage in the limbic system involving these ACh neurons. In detail, damage to septal ACh neurons was recently reported in a parkinsonian syndrome with a severe course such as progressive supranuclear palsy [112]. Similarly, the loss of MSN characterizes mixed PD/Alzheimer’s disease (AD) degeneration [113]. This is backed up by preclinical findings showing that pharmacological manipulation of ACh MSN neurons may rescue the failure in working memory, which develops in toxic models of PD [114]. Finally, in PD patients, cortical connectivity of basal forebrain ACh neurons is significantly altered, posing these ACh neurons as the candidate to produce cognitive dysfunction in PD-related degenerative disorders [115]. Damage to septal nuclei in PD may underlie the loss of cognitive functions occurring in PD patients and in patients affected by other phenotypes of parkinsonism (such as dementia with Lewy bodies, DLB). On the other hand, recent studies place the MSN at center stage in producing a whole *continuum* of symptoms occurring in PD patients. In fact, it is demonstrated that thalamic innervation as well as descending projections from the MSN to the brainstem reticular formation may have a significant effect on movement control and adjusting correct muscle tone and body posture [116]. Thus, both motor and non-motor symptoms may be significantly impacted by a loss of MSN ACh neurons during parkinsonism. This is not unusual considering that the MSN is an integral part of the basal ganglia, and it is often referred to as the ventral (limbic) striatum, which is massively involved in PD [116]. The basal ganglia, thalamic, and brainstem circuitry of this nucleus place it at a strategic crossroads to affect cortical motor circuitry, cognition, and ancestral motor control, including muscle tone and posture. It may be useful to recall that this nucleus also receives a powerful DA innervation from the mesolimbic pathway arising from the para-nigral nucleus (ventral tegmental area, VTA, A10), and it is affected at certain stages in the disease course of degenerative parkinsonism [117]. This is in line with recent magnetic resonance imaging (MRI) data obtained from 80 PD patients where, following manual segmentation of the MSN, a decrease in nuclear volume was significantly documented [116]. These results need to be emphasized when considering the relative preservation of connected neighboring limbic regions. Such a distinct impairment of the MSN in PD was often underestimated in previous classic studies, while it appears to be strongly associated with motor impairment. The role of an altered DA innervation in producing MSN degeneration is unlikely when considering the results of the present study, where pure MSN neurons deprived of their DA connections are markedly sensitive to MPP^+^. This is probably why the volume loss within MSN is suggested as an early biomarker to assess patients at risk of developing PD [116]. Although the present study provides evidence that sensitivity of MSN ACh neurons to MPP^+^ is more pronounced than in immortalized catecholamine (DA/NE) cell lines, the reason for such a difference remains unknown nowadays. Therefore, considering the present data, it is no longer surprising that the original findings by Lewy described “Lewy bodies” within *Substantia Innominata* (corresponding to the ACh nucleus Ch4) from PD patients rather than in the SN. On the other hand, it is quite frequent that patients suffering from PD with dementia or DLB feature a marked neuronal loss, which extends beyond the SN to recruit the para-nigral DA cell group and basal forebrain ACh neurons [50,118], suggesting a dysfunction in cortical ACh pathways before frank neuronal degeneration occurs [119]. In this latter study, patients with PD and dementia (often being diagnosed as affected by DLB) were differentiated by a significant reduction in hippocampal ACh activity, by a significant loss of non-pigmented lateral A10 DA neurons and Ch4 ACh neurons (30% and 55% cell loss, respectively, compared with neuronal preservation in control subjects). The significance of a dysfunction of the MSN in PD may also extend to other non-motor symptoms, such as psychotic symptoms as suggested by the beneficial effects of deep brain stimulation of the medial septum in patients affected by PD to treat psychotic symptoms effectively [120]. This recruitment of MSN along with other ACh cell groups in the basal forebrain may appear to be odd at first glance within the frame of PD as a disorder typically affecting catecholamine neurons of the brainstem (such as SN and LC). On the other hand, when considering the cytomorphology of ACh nuclei and their authentic significance, they reveal an impressive commonality with classic reticular brainstem nuclei [121]. In fact, as reported in the pioneer paper by Arendt and colleagues [121], ACh nuclei are the rostral border of the core of the reticular formation as witnessed by their isodendritic, reticular cytomorphology and widespread connections [122]. In light of redefining PD as a brainstem reticular monoamine disorder [123] rather than a pure DA disorder, several reticular nuclei are reasonably involved, and this applies mostly to the archaic *core* of the reticular formation. These nuclei are collectively affected during PD, extending to AD, DLB, and other degenerative dementias such as Korsakoff’s disease. It is remarkable that, during neurodegeneration, these nuclei undergo plasticity, which may be interpreted as a compensatory mechanism. In the present manuscript, augmented plasticity was described following administration of curcumin in damaged MSN ACh cells following MPP^+^. In detail, neuronal branching is increased as much as described for MSN by the classic study of Arendt and colleagues [121]. This latter manuscript was the first to describe dendritic reorganization within MSN when neuronal loss occurred in PD, AD, and in ageing. This phenomenon is extended to the whole class of open nuclei receiving infra-modal connections from the whole brain axis, which are all affected in neurodegenerative disorders and form the archaic core of the reticular formation (SN, VTA, LC, Ch1, Ch2, Ch3, Ch4) [122]. The protective effects of curcumin are remarkable when considering the amount of neuroprotection and the mechanisms involved. In detail, evidence provided here by the Bafilomycin A1 assay is consistent with the impairment of the autophagy flux by MPP^+^, which appears to be reversed by curcumin. This is in line with expression levels of key autophagy proteins such as LC3-II and p62, which are assessed following each treatment. Similarly, the quantitative measurement of PINK1 and Parkin, in situ by TEM combined with immunogold staining, is compatible with this interpretation. While MPP+-induced mitochondrial damage triggers PINK1/Parkin expression on mitochondria, the addition of curcumin removes PINK1/Parkin positive mitochondria, consistent with promoting autophagy/mitophagy. All these data suggest that the progression of autophagy vacuoles stained with the active, lipidated isoform LC3-II and the potential recruitment of mitophagy (mitochondrial removal by autophagy), leading to a decrease in PINK1- and Parkin-positive mitochondria, is promoted by curcumin. This hypothesis is supported by the bafilomycin assay. The effects of curcumin may also consist of some neurorescue. However, caution is needed when interpreting these effects of curcumin administered at early time intervals. In detail, data indicate that curcumin, apart from protecting when administered before MPP^+^, alters the progression of cell death from 24 to 72 h when it is administered at 24 h following MPP^+^. These effects can be cautiously defined as disease-modifying effects rather than neurorescue. Indeed, these latter findings do not tell whether curcumin is neurorescuing or neuroprotectant or both. If curcumin were to play a mere protective effect, cell degeneration would not move back towards control values following the addition of curcumin at 24 h after MPP^+^. Instead, curcumin modifies the progression of cell degeneration, which may occur through different effects. The present data indicate that curcumin is likely to exert beneficial effects both at early (a few hours after) and delayed (up to 72 h) time intervals following MPP^+^.

### Limitations of the Study and Future Research Directions

When interpreting the present data, one should always keep in mind the biological matrix being used. This is represented by in vitro cells derived from the immortalization of ACh MSN. Although the ubiquitous phenotype of these cells was validated in the present study by the expression of ACh-related antigen within the whole cell population (Supplementary Figure S1), the present findings are inherently limited in connection with the lack of afferent systems regulating the activity of medial septal neurons (NE axons from LC, DA axons from the VTA, 5-hydroxytryptamine (5-HT) axons from the dorsal raphe nucleus, and glutamate axons from the limbic cortex and hippocampus, mainly the *Cornu Ammonis*). Therefore, the vulnerability of these neurons in vivo is expected to possess some potential divergence compared with data in vitro. Nonetheless, in previous and unique work carried out by administering MPTP to C57Bl mice, a marked vulnerability of cholinergic neurons in the medial septum was reported [50]. This latter evidence strengthens the significance of the present exploration carried out at the cellular level of which targets and protective mechanisms operate within this brain area. Future research directions are expected to clarify whether curcumin exerts a pure protective effect, or it combines protection and rescue. This may explain why the post-treatment of curcumin halts the progression of the damage in the time window between 24 h and 72 h. Ad hoc experiments may help clarify this effect from the perspective of administering autophagy-inducing compounds once clinical symptoms already exist.

Finally, a future research direction, which is dictated by the present results, concerns the ability of curcumin to be absorbed following ingestion [124] and to cross the blood–brain barrier [125]. Since in vitro studies cannot predict these pharmacokinetics issues, experiments designed to assess and overcome the limitations of curcumin bioavailability are needed [124,125]. Future research is expected to design nano-vectors or chemically coordinated molecular groups to improve the delivery of curcumin within the CNS. The present study highlights the higher vulnerability of ACh neurons compared with DA neurons to MPP^+^. This is a novelty in MPP^+^ research. So far, catecholamine-containing cells were the most sensitive to MPP^+^, which was attributed to the quite selective uptake of MPP^+^ through the DAT/NET transporters. The mechanisms of entry of MPP^+^ into ACh neurons need to be established, as well as the specific cellular mechanism affected within these neurons. It is likely that mitochondria remain the main target, as shown in the present study, although additional targets may be involved as well. For instance, ACh release, which occurs following MPP^+,^ may produce calcium-mediated damage through nicotinic receptors, or muscarinic receptors may be involved through the activation of cell death pathways. All these features represent an issue to be addressed during future research activities.

## 4. Materials and Methods

### 4.1. Cell Cultures

SN56 ACh cell culture consists of ACh septal neurons, which are obtained from ACh neurons dissected from the *septum pellucidum* of C57BL/6 mice at postnatal day 21. These neurons were immortalized through fusion with N18TG2 neuroblastoma cells [52,53]. This cell culture is not commercially available. We obtained these cells from Prof. Laura Calzà (University of Bologna, Bologna, Italy), who kindly provided aliquots of the cell line that had been cryopreserved and maintained in her laboratory. Cells were cultured in DMEM medium (Sigma-Aldrich, St. Louis, MO, USA) containing 2 mM L-Glutamine and supplemented with 10% fetal bovine serum (FBS, Sigma) and 0.1% penicillin (50 IU/mL)/streptomycin (50 μg/mL, from a stock solution of 50 mg/mL, Sigma-Aldrich). The culture was maintained under standard conditions at 37 °C and 5% CO_2_.

PC12 catecholaminergic cell line was purchased from a cell bank (IRCCS San Martino Institute, Genova, Italy). Cells were grown in RPMI 1640 medium (Sigma-Aldrich), supplemented with heat-inactivated 10% horse serum (Sigma-Aldrich), 5% fetal bovine serum (Sigma-Aldrich), and penicillin (50 IU/mL)/streptomycin (50 μg/mL, Sigma-Aldrich). The cell culture was kept under standard conditions in a humidified atmosphere at 37 °C and 5% CO_2_.

All experiments were carried out during the log-phase of cell growth, corresponding to roughly 70% confluence [126,127].

For TB staining experiments, both SN56 and PC12 cells were seeded at a density of 1 × 10^4^ cells/well within 24-well plates in 1 mL of culture medium.

For light microscopy experiments, 5 × 10^4^ SN56 and PC12 cells were seeded on coverslips, which were placed in 24-well plates with 1 mL/well of culture medium.

For electron microscopy experiments, 1 × 10^6^ SN56 cells were seeded in 6-well plates with 2 mL/well of culture medium.

### 4.2. Experimental Design

The present study was designed to assess whether MPP^+^-induced damage to ACh neurons may be produced directly by the effects of MPP^+^ on vulnerable MSN ACh neurons. Although some findings report that, following MPTP, a loss of ACh neurons in the pro-encephalon takes place [49,50,51], these in vivo approaches leave open the question of whether this is the consequence of damage produced by a loss of DA/NE function or whether MPTP directly destroys MSN ACh neurons through its active metabolite MPP^+^. Thus, the present study was carried out by profiting from ad-hoc neurons developed from the C57BL/6 mouse MSN from postnatal day 21, which were previously immortalized through a fusion with the parent neuroblastoma N18TG2 cells [52,53]. The toxic effects of MPP^+^ on MSN ACh neurons were characterized by drawing a dose-dependent curve using increasing doses of MPP^+^ (ranging from 0.1 μM up to 500 μM) for 72 h. Neurotoxicity was assessed by light microscopy with histochemical staining aimed at detecting degenerating neurons, which were followed by ultrastructural studies at TEM mostly focused on detecting the occurrence of mitochondrial damage. Since specific phytochemicals, such as curcumin, are claimed to produce protective effects in neurodegenerative disorders, curcumin was administered at a dose known to protect catecholamine neurons against methamphetamine toxicity (10 μM, [62]). In detail, curcumin was administered either before (to test neuroprotection) or after (to test potential neurorescue/disease-modifying effect) MPP^+^. Concomitantly, curcumin was analysed for the potential preservation of subcellular pathology operating in the cells and plastic events counteracting mitochondrial alterations. This experimental hypothesis was grounded on previous studies showing that curcumin protects catecholamine (including DA) cell lines against toxins like MPP^+^ and MPTP by preventing mitochondrial dysfunction [71,72,73].

### 4.3. Cell Treatments

A stock solution of 10 mM MPP^+^ was prepared by dissolving 3.0 mg of MPP^+^ in 1 mL of culture medium. The final working solutions were obtained by diluting appropriate aliquots of the stock solution in the culture medium.

In the first part of this study, the effects of MPP^+^ on SN56 cell viability were evaluated. In detail, a dose–response curve to evaluate the toxic effects of MPP^+^ on SN56 cells was carried out by treating the cell culture with increasing doses of MPP^+^ (0.1, 1, 10, 100 and 500 μM) for 72 h. This allowed us to select the MPP^+^ dose able to produce moderate toxicity, which corresponded to 10 μM. This MPP^+^ dose was used in the experiments carried out in the second part of the study to investigate the potential neuroprotection/neurorescue/disease-modifying effects of curcumin.

To compare the toxicity induced by MPP^+^ in ACh cells with that produced in catecholamine-containing cells, the dose-dependent effects of MPP^+^ on cell viability were also evaluated in the catecholaminergic PC12 cell line. PC12 cells were treated with increasing concentrations of MPP^+^ (0.1–500 μM) for 72 h, following the same dose–response protocol used for SN56 ACh cells.

In the second part of our study, SN56 cells were treated with a curcumin dose of 10 μM for 72 h, which was selected based on a dose–response curve that was carried out by treating SN56 cells with increasing doses of curcumin, ranging from 0.1 to 50 μM, for 72 h. The safety of such a curcumin dose confirms our previous in vitro studies carried out in other cell cultures [62,65,74,75].

A stock solution of curcumin 9.5 mM was prepared by dissolving 3.5 mg of curcumin powder (Product Number C7727, CAS 458-37-7, purity ≥ 80% in curcumin and ≥94% in total curcuminoids, Sigma-Aldrich) in 1 mL of DMSO (Sigma-Aldrich). The curcumin doses used in the experiments were obtained by diluting an aliquot of the stock solution in the culture medium.

In combined experiments, curcumin was administered either 2 h before MPP^+^ (to test neuroprotection) or 24 h following MPP^+^ (to test neurorescue/disease-modifying effects). Control cells were treated with vehicle, consisting of culture medium containing 0.1% DMSO. In preliminary experiments, the safety of DMSO at a concentration up to 0.5% (which is the DMSO concentration within the 50 μM curcumin dose used in dose-dependent experiments) was assessed.

### 4.4. The Bafilomycin A1 Autophagy-Flux Assay

In experiments aimed at assessing the autophagy flux, the autophagy inhibitor bafilomycin A1 (100 nM, Sigma-Aldrich) was added to the culture medium 3 h before the end of the treatments with MPP^+^ or curcumin. In detail, bafilomycin A1 disables lysosomes since it is a powerful and specific inhibitor of vacuolar-type H^+^-ATPases, and impairs the fusion of autophagosomes with lysosomes. In this way, the autophagy flux can no longer proceed towards the final lysosomal clearance. Thus, adding bafilomycin impairs the enzymatic degradation of both LC3-II and p62. Bafilomycin is useful to assess the autophagy status by comparing the sample in the presence vs. the absence of bafilomycin to interpret whether the increase in LC3-II may be due to autophagy activation or inhibition. When autophagy is active, LC3-II increases (degradation of LC3-II is balanced by increased synthesis of LC3-II, while p62 decreases due to autophagy degradation). In this condition, adding bafilomycin increases both LC3-II and p62. Still, conclusions need to be cautiously interpreted.

### 4.5. Hematoxylin and Eosin (H&E) Histochemistry

At the end of the treatments, SN56 cells were exposed to paraformaldehyde 4%in PBS for 15 min, then washed in PBS and immersed in hematoxylin solution (Sigma-Aldrich) for 20 min. Subsequently, slides were washed out and plunged into eosin (Sigma-Aldrich) for 20 s. The excess dye was removed by several washings, and cell dehydration was carried out by plunging the dish in serial ethanol, finally cleared in xylene. Stained slides were collected and layered with DPX medium (Sigma-Aldrich). Cells were counted using a Nikon Eclipse 80i light microscope (Nikon, Tokyo, Japan) at 20× magnification within three microscopic fields, where only distinct cells that were not overlapping were considered in the count. Data were validated by repeating the experiments three times, independently.

### 4.6. Trypan Blue (TB) Staining

After treatments, cells were collected and centrifuged at 800× *g* 5 min. The cell pellet was suspended in 0.5 mL of the original culture medium, and 25 μL of cell suspension was added to a 62.5 μL solution containing 1% TB (Sigma-Aldrich) and 37.5 μL of PBS, for 5 min, at room temperature. Soon after, 10 μL of this solution was transferred into a Bürker glass chamber, and both total cells and TB-positive cells were counted. The TB method is based on the fact that TB penetrates non-viable cells through a damaged membrane [128,129,130]. Cell count was carried out using an Olympus CKX 41 inverted microscope (Olympus Corporation, Tokyo, Japan). Data were validated in three independent experiments. In each experiment, the mean percentage ± S.E.M. of TB-positive cells was calculated.

### 4.7. FluoroJade-B (FJ-B) Histofluorescence

PBS-washed cells were fixed in 4% paraformaldehyde for 5 min. Then, cells were washed in PBS, and they were incubated in 0.06% potassium permanganate for 10 min at room temperature. After washing in distilled water, cells were incubated for 20 min in a 0.0004% FJ-B solution. The principle of FJ-B consists of the selective binding of degeneration-dependent polyamines to the fluorescent dye, allowing staining of degenerating cells [76,77,78]. FJ-B solution is prepared from a 0.01% FJ-B stock solution (Merck Millipore, Billerica, MA, USA) diluted in distilled water containing 0.1% acetic acid. Following washing, cells are mounted using DPX medium (Sigma-Aldrich). Cell count is carried out using a Nikon Eclipse Ni light microscope, equipped with a fluorescence lamp (Nikon, Tokyo, Japan) at 20× magnification within three microscopic fields. Distinct cells were counted. The measurement of fluorescence was based on a cutoff intensity according to previous studies [131]. Optical density obtained under a fluorescence microscope was calculated through ImageJ software (NIH, Bethesda, MD, USA, Version 1.8.0_172).

### 4.8. Mitochondrial Labelling

To stain healthy mitochondria cells were incubated in a solution of MTR-R = healthy (Thermo-Fisher Scientific, Waltham, MA, USA), or MTR-G = total (Thermo-Fisher Scientific), which labels total cell mitochondria [79,80,81]. The different staining of MTR-G = total compared with MTR-R = healthy is due to the mitochondrial retention of the dye, which is independent of alterations of the membrane potential for MTR-G = total, while MTR-R staining is lost when mitochondrial membrane potential is altered, which explains why MTR-R stains healthy mitochondria only. Both MTR-G = total and MTR-R = healthy were diluted to reach a final concentration of 500 nM for 45 min, at 37 °C and 5% CO_2_. MTR-R = healthy or MTR-G = total solutions were removed, and cells were analysed according to previous studies [131] by using ImageJ software (NIH, USA, Version 1.8.0x172).

### 4.9. Immunohistochemistry (Immunofluorescence and Immunoperoxidase)

At the end of the treatments, cells were washed in PBS and fixed with 4% paraformaldehyde in PBS for 15 min, and then they were incubated in 0.1% TritonX-100 for 15 min in PBS.

For immunofluorescence experiments, cells were washed and immersed in PBS containing 10% normal goat serum for 1 h at room temperature (RT) to block aspecific antigen sites and finally incubated overnight at 4 °C in 1% normal goat serum in PBS containing the following primary antibodies: (i) rabbit anti-LC3-II (2775, Cell Signalling Technology, Danvers, MA, USA) (ii) mouse anti-PINK1 (ab186303, Abcam, Cambridge, UK); (iii) rabbit anti-Parkin (AB5112, Millipore, Burlington, MA, USA); (iv) mouse anti-p62 (MA5-31498, Invitrogen, Carlsbad, CA, USA). All primary antibodies were diluted 1:100. After rinsing in PBS, cells were incubated at RT for 1 h with the appropriate fluorophore-conjugated secondary antibodies Alexa 488 (A11008, green fluorophore, Life Technologies, Carlsbad, CA, USA) or Alexa 546 (A11003, red fluorophore, Life Technologies), both diluted 1:200. After washing in PBS, nuclei were stained by the fluorescent dye DAPI (Sigma-Aldrich), which was added at a dilution of 1:1000 in distilled water for 5 min. Then cells were washed in PBS, transferred onto a coverslip, mounted with the mounting medium Fluoroshield (Sigma-Aldrich, Saint Louis, MO, USA), and observed under the Nikon Eclipse 80i light microscope (Nikon) equipped with a fluorescent lamp and a digital camera connected to the NIS Elements Software for image analysis (Nikon). Negative control cells were incubated with secondary antibodies only. Double-stained pictures were acquired independently and then merged. The measurement of immunofluorescence was based on a cutoff intensity according to Marwaha and Sharma [131]. Optical density of immunofluorescence as well as the immunofluorescent areas were measured under a fluorescence microscope using ImageJ software (NIH, MD, USA, Version 1.8.0_172).

For immuno-peroxidase experiments, cells were washed and then incubated in 3% hydrogen peroxide for 20 min at RT to block endogenous peroxidase activity. Subsequently, they were plunged in a blocking solution containing 10% NGS in PBS for 1 h at RT. Cells were incubated overnight at 4 °C with the primary antibody solution containing 2% NGS in PBS and the rabbit anti-ChAT antibody (ab178850, Abcam) diluted 1:100.

The antigen–antibody reaction was revealed using the anti-rabbit biotin-conjugated secondary antibody (BA-1000, Vector Laboratories, Newark, CA, USA) diluted 1:200 for 1 h at RT, which was followed by incubation with the avidin-biotin complex (Vector Laboratories) for 1 h and the peroxidase substrate diaminobenzidine (Vector Laboratories) for 7–8 min. Then, cells were dehydrated using increasing alcohol solutions. After washing in PBS, cells were cleared in xylene, gently transferred onto cover slips, and DPX mounting medium (Sigma-Aldrich) was added. Finally, cells were observed by light microscopy (Nikon) equipped with a digital camera connected to the NIS-Elements software (NIS-Elements D 5.30.00 build 1531, 64-bit) for image analysis (Nikon, Tokyo, Japan). Negative control cells were incubated with the secondary antibody solution only.

### 4.10. Transmission Electron Microscopy (TEM)

Samples were fixed using a mixture of 2.0% paraformaldehyde and 0.1% glutaraldehyde, which was added for 90 min at 4 °C. Fixed cells were scraped from the dish and centrifuged at 10,000 rpm for 10 min. After washing, cell pellets were post-fixed in 1% osmium tetroxide (OsO_4_) for 1 h at 4 °C. Cells were dehydrated through serial ethyl alcohol solutions, and they were embedded in epoxy resin. The use of this published protocol allows minimal epitope coverage and maintains cell architecture [132].

Samples were reduced to ultrathin slices (90 nm thick), which were obtained from an ultra-microtome (Leica Microsystems, Wetzlar, Germany). These slices were exposed to a saturated solution of uranyl acetate and lead citrate. Stained slices were analyzed through a JEOL JEM SX100 transmission electron microscope (JEOL, Tokyo, Japan).

### 4.11. Ultrastructural Morphometry of Mitochondria

Non-serial ultrathin slices were mounted on grids to be magnified at 6000×. At least 50 cells per experimental group were analysed concerning the mitochondrial status in three independent experiments. Total and altered mitochondria were counted in each cell. The count was systematic, starting from a randomized corner; thus, a whole scanning of the cell pellet was carried out through equally spaced parallel sweeps. The structure of mitochondria (double membrane with intermembrane space; the labyrinth-like system delimited by the inner membrane; the homogeneous matrix and quite regularly intermingled cristae) was identified according to well-established criteria [133,134].

Counts of mitochondria were made at 8000×. Mitochondrial alterations were defined according to our previous studies [135], which defined the criteria to classify a mitochondrion as altered. The number of altered mitochondria was calculated following MPP^+^ with or without curcumin administration compared with controls; each experiment was validated to obtain a total of three independent assessments.

### 4.12. Post-Embedding Immunogold Electron Microscopy

Ultrathin slices were collected on nickel grids and immersed for 30 min in aqueous sodium metaperiodate, at RT, to remove OsO_4_. After repeated washing in PBS, grids were placed on droplets of a PBS 1 M blocking solution containing 10% goat serum and 0.2% saponin for 20 min at RT to prevent non-specific staining. Then, grids were incubated at 4 °C overnight in the primary antibody solution, containing 1% goat serum, 0.2% saponin, and primary antibodies in ice-cold PBS. In detail, the primary antibodies used were the following: (i) rabbit anti-LC3-II (2775, Cell Signalling Technology, Danvers, MA, USA); (ii) mouse anti-PINK1 (ab186303, Abcam); (iii) rabbit anti-Parkin (AB5112, Millipore, Burlington, MA, USA). All primary antibodies were diluted 1:50.

At the end of the incubation, grids were washed in cold PBS (three times, 5 min each) and they were placed for 1 h at RT on drops of a PBS solution containing 1% goat serum, 0.2% saponin and gold-conjugated (10 nm or 20 nm gold particles) secondary antibodies diluted 1:100. In detail, anti-mouse gold-conjugated secondary antibodies (10 nm gold particles, EM.GMHL10, BB International, Cardiff, UK) and anti-rabbit gold-conjugated secondary antibodies (20 nm gold particles, EM.GAR20, BB International) were used. Control ultrathin sections were incubated with secondary antibodies only. In addition, negative controls were checked for secondary antibody by incubating samples with primary antibody only, without incubation with immunogold-conjugated secondary antibodies.

Grids were washed in PBS (three times, 5 min each) and placed for 3 min on drops containing 1% glutaraldehyde in PBS. Then, after washing in distilled water, grids were stained with uranyl acetate and lead citrate, and ultrathin sections were finally observed on TEM (JEOL JEM SX100).

Counts at TEM were carried out using a magnification of 8000×, which allows detection of immunogold particles within specific subcellular compartments.

### 4.13. Statistical Analysis

To count cell viability, cells were randomly selected. Thus, we neither referred to nor selected any specific area of the slide for the count. Rather, to document cell viability, quantitative measurements of the number of H&E- and FJ-B-positive cells were carried out under light microscopy within different microscopic fields at 20× magnification, allowing analysis of an area of about 6.3 × 10^5^ μm^2^. Microscopic fields were randomly selected from each experimental group. Cell counts were carried out by blind researchers. Values are expressed as the mean percentage ± S.E.M. of H&E-stained cells counted from each experimental group, assuming controls equal to 100%, or as the mean number ± S.E.M. of FJ-B-fluorescent cells counted from each experimental group. The number of damaged/non viable cells was also measured by counting TB-positive cells. This was carried out under the inverted microscope, where both TB-positive and TB-negative cells are visible. In detail, TB serves as an indicator of dead cells since, under baseline conditions, viable cells are not permeable to TB, while TB easily enters dead cells through damaged membranes. Although the irreversibility of this process remains debatable, it is generally assumed that TB added to a cell culture selectively stains dead cells [128,129,130]. Data are expressed as mean percentage ± S.E.M. of TB-positive cells out of the total number of cells. Finally, degenerating cells were measured by counting cells stained by FJ-B, a fluorescent dye that binds specific cytosolic polyamines, which increase during a short time window (24 h–72 h) within dying cells [76,77,78]. This method provides a yellow fluorescent staining based on the chemical nature of its anionic fluorescein derivative used for the histological staining of neurons undergoing degeneration. In this case, data are expressed as mean ± S.E.M. of FJ-B-fluorescent cells and as mean percentage ± S.E.M. of FJ-B fluorescence (assuming controls = 100%). To validate the statistical significance, all experiments were carried out three times independently, and consistent results were obtained within each experiment. Moreover, three different methods were used to assess cell viability, which provides internal validation of the data, since apart from slight discrepancies (due to differences in the specific procedures), the consistency of the results obtained using different techniques strengthens the assessment of neuronal damage.

Cells used for the measurement of the mean fluorescence intensity and the mean flu-orescent area, along with the mean number and the mean length of cell branching at light microscopy, were 30 cells from each group. Each experiment was repeated three times in-dependently to validate the significance of differences obtained between groups in each independent experiment. This means that fully independent experiments were carried out with n = 30 cells from each group. The number of cell branches (synonymous with cell processes) was measured using a threshold of 0.1 μm length to consider an authentic cell branch. The mean number ± S.E.M. of these branches per cell was expressed for each group. In addition, the longest branch in each cell was counted, and the mean length ± S.E.M. of the longest process per cell was expressed following the count in 30 cells from each experimental group.

For immunofluorescence, values are expressed as the mean percentage ± S.E.M. of both fluorescence intensity and fluorescent area (assuming controls = 100%), measured in n = 30 cells from each experimental group. Each experiment was carried out three times (triplicate) to assess consistency of data in independent experimental conditions.

The count of total and altered mitochondrial number, and the in situ stoichiometry assessment of several antigens (i.e., LC3-II, PINK1, Parkin), were carried out by TEM in 16–17 cells from each experimental group. Each independent experiment was repeated three times. Data represent the mean ± S.E.M. of 16–17 cells per experimental group. Each experiment was repeated three times with comparable data concerning statistical inference from each independent experiment. Measurement of the mitochondrial area was carried out in n = 10 cells per group, and it was repeated again in three independent experiments. Values are expressed as the mean number ± S.E.M. or the mean percentage ± S.E.M. (assuming controls = 100%).

In each independent experiment, comparisons among different groups were carried out by one-way analysis of variance (ANOVA), followed by Scheffé’s post-hoc analysis. The null hypothesis (H0) was rejected for *p* < 0.05.

Data from each count from each of the three independent experiments will be made available as raw data upon request.

## Figures and Tables

**Table 1:** Length of cell processes.

	Controls	MPP^+^	Curcumin	Curcumin + MPP^+^	MPP^+^ + Curcumin
Length (μm)	0.3 ± 0.05	0.7 ± 0.1	16.4 ± 1.5 °	7.0 ± 1.3 °	6.2 ± 0.1 °

The mean length ± S.E.M. of the longest cell process per cell is reported. ° *p* < 0.05 compared with controls and MPP^+^.

## Data Availability

The raw data supporting the conclusions of this article will be made available by the authors on request.

## Supplementary Materials

The following supporting information can be downloaded at: https://www.mdpi.com/article/10.3390/molecules31213814/s1, Figure S1: ChAT immunohistochemistry in SN56 cells; Figure S2: Sensitivity of catecholaminergic cells to MPP^+^ toxicity; Figure S3: Dose-dependent effects of curcumin on cell viability; Figure S4: Cell viability after exposure to DMSO; Figure S5: Opposite effects of MPP+ and curcumin on mitochondrial area; Figure S6: MPP+ decreases while curcumin increases autophagy flux; Figure S7: MPP+ increases, while curcumin decreases, PINK1- and Parkin-positive mitochondria; Figure S8: Opposite effects of MPP+ and curcumin on LC3-II intracellular compartmentalization.

## Abbreviations

The following abbreviations are used in this manuscript:

5-HT	5-Hydroxytryptamine
ACh	Cholinergic
AD	Alzheimer’s Disease
Ch1-Ch4	Basal forebrain cholinergic nuclei
DA	Dopamine
DLB	Dementia with Lewy bodies
FJ-B	Fluoro Jade-B
H&E	Hematoxylin & Eosin
LC3-II	Lipidated microtubule-associated protein 1A/1B-light chain 3
LC	Locus coeruleus
MPP^+^	1-methyl-4-phenyl pyridinium
MPTP	1-methyl-4-phenyl-1,2,3,6-tetrahydropyridine
MSN	Medial septal nucleus
MTR-G	MitoTracker Green
MTR-R	MitoTracker Red
NE	Norepinephrine
PD	Parkinson’s Disease
SN	Substantia nigra
TB	Trypan Blue
TEM	Transmission Electron Microscopy
VTA	Ventral Tegmental Area
