# Supplementary material for: Curcumin Prevents Degeneration of Medial Septal Cholinergic Neurons Produced by Parkinsonism-Inducing Neurotoxin 1-Methyl-4-Phenylpyridinium
Source: Molecules. 2026 Oct 22;31(21):3814. doi: 10.3390/molecules31213814 (PMC13764732; doi:10.3390/molecules31213814)
Supplement: Supplementary file 1 [file molecules-31-03814-s001.zip › molecules-4500199-supplementary.pdf]

## Supplementary Figures

ChAT

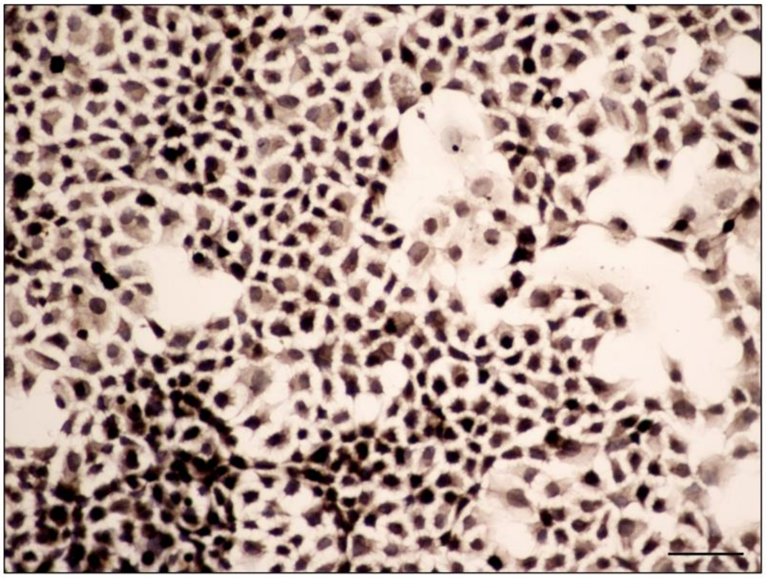

**Supplementary Figure S1.** ChAT immunohistochemistry in SN56 cells. The representative picture of control SN56 cells following immunohistochemistry for the specific ACh marker ChAT shows the ubiquitous expression of the protein in the whole cell culture. ChAT=choline acetyltransferase. Scale bar=58 µm.

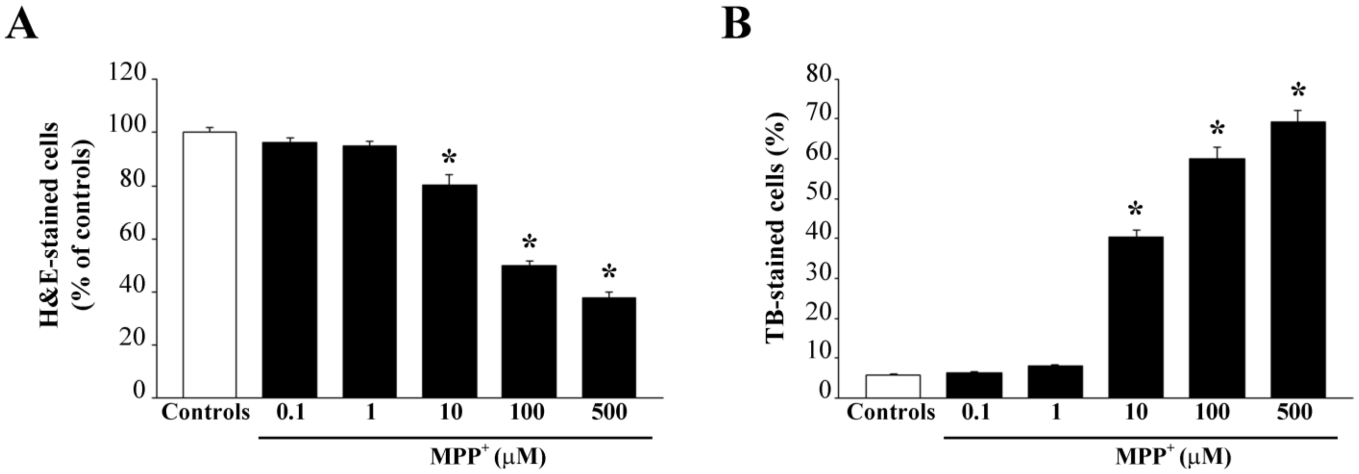


**Supplementary Figure S2.** Sensitivity of catecholaminergic cells to $MPP^+$ toxicity. Dose–response curves for cell viability following exposure to increasing concentrations of $MPP^+$ (0.1–500 μM) in PC12 cells, assessed by H&E (**A**) and TB (**B**) staining. Catecholaminergic cells were approximately ten-folds less sensitive to $MPP^+$-induced toxicity than Ach cells. *P<0.05 compared with controls.

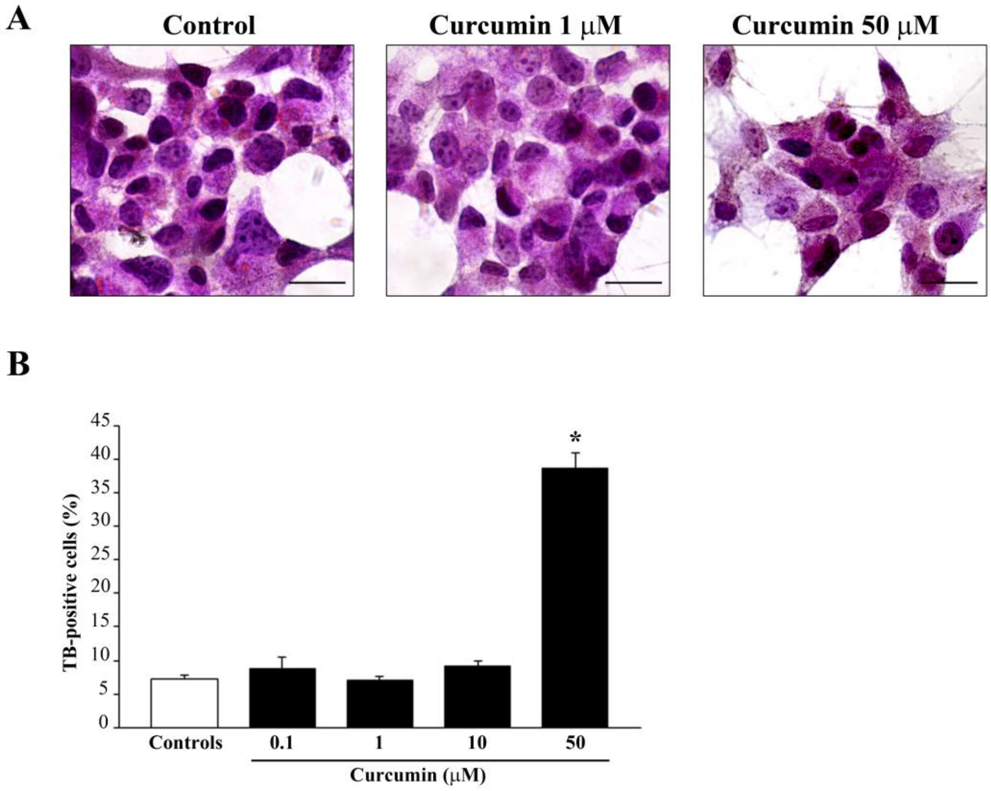


**Supplementary Figure S3.** Dose-dependent effects of curcumin on cell viability. Cell viability of SN56 ACh neurons exposed to increasing doses of curcumin, ranging from 0.1 to 50 μM, was measured through the TB staining assay. (**A**) Representative H&E-stained cells and (**B**) the graph reporting the percentage of TB-positive cells out of total cells indicate that curcumin doses up to 10 μM are safe for SN56 ACh neurons, while the curcumin dose of 50 μM affects cell viability. *$P<0.05$ compared with controls. Scale bars=22 μm.

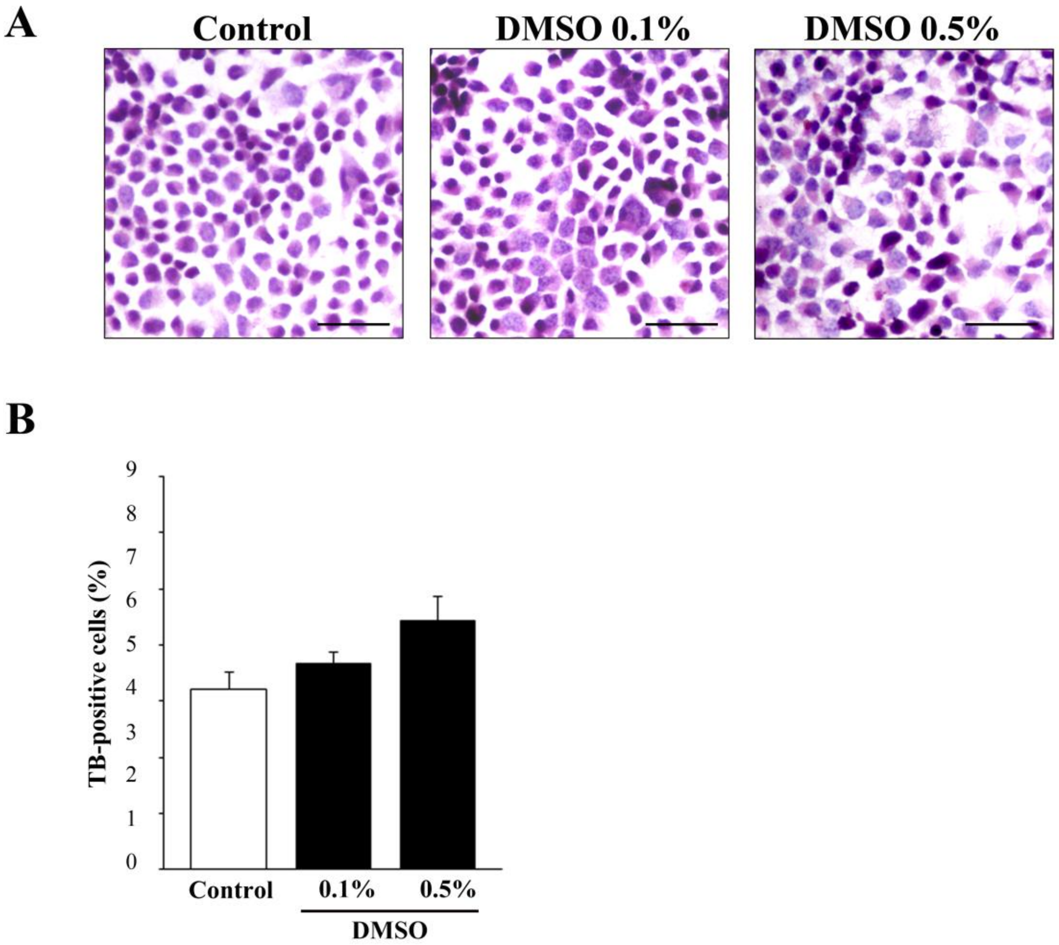


**Supplementary Figure S4.** Cell viability after exposure to DMSO. Cell viability of SN56 ACh neurons exposed to DMSO concentrations of 0.1% and 0.5%, which are used in this study. (**A**) Representative H&E-stained cells and (**B**) the graph reporting the percentage of TB-positive cells out of total cells indicate that these DMSO concentrations do not affect viability of SN56 ACh neurons. Scale bars=58 μm.

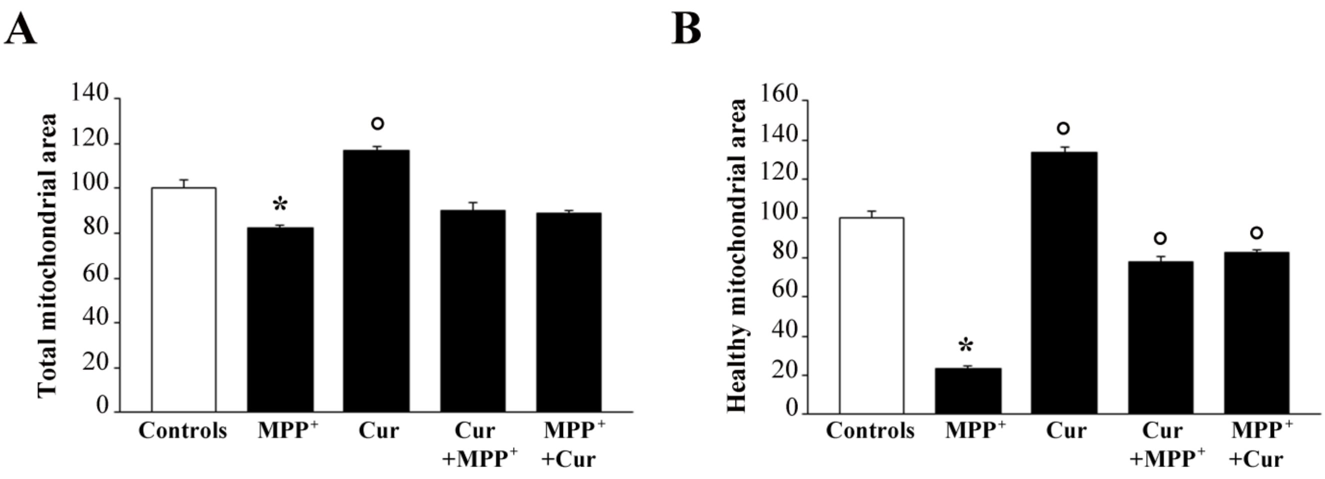


**Supplementary Figure S5.** Opposite effects of $MPP^+$ and curcumin on mitochondrial area. Graphs report the measurement of the (**A**) total mitochondrial area, and (**B**) healthy mitochondrial area, form control cells and cells treated with $MPP^+$, curcumin and combined treatment with curcumin and $MPP^+$, where curcumin is administered before (Cur+$MPP^+$) or after ($MPP^+$+Curcumin) $MPP^+$. Cur=curcumin. *$P<0.05$ compared with controls; °$P<0.05$ compared with controls and $MPP^+$.

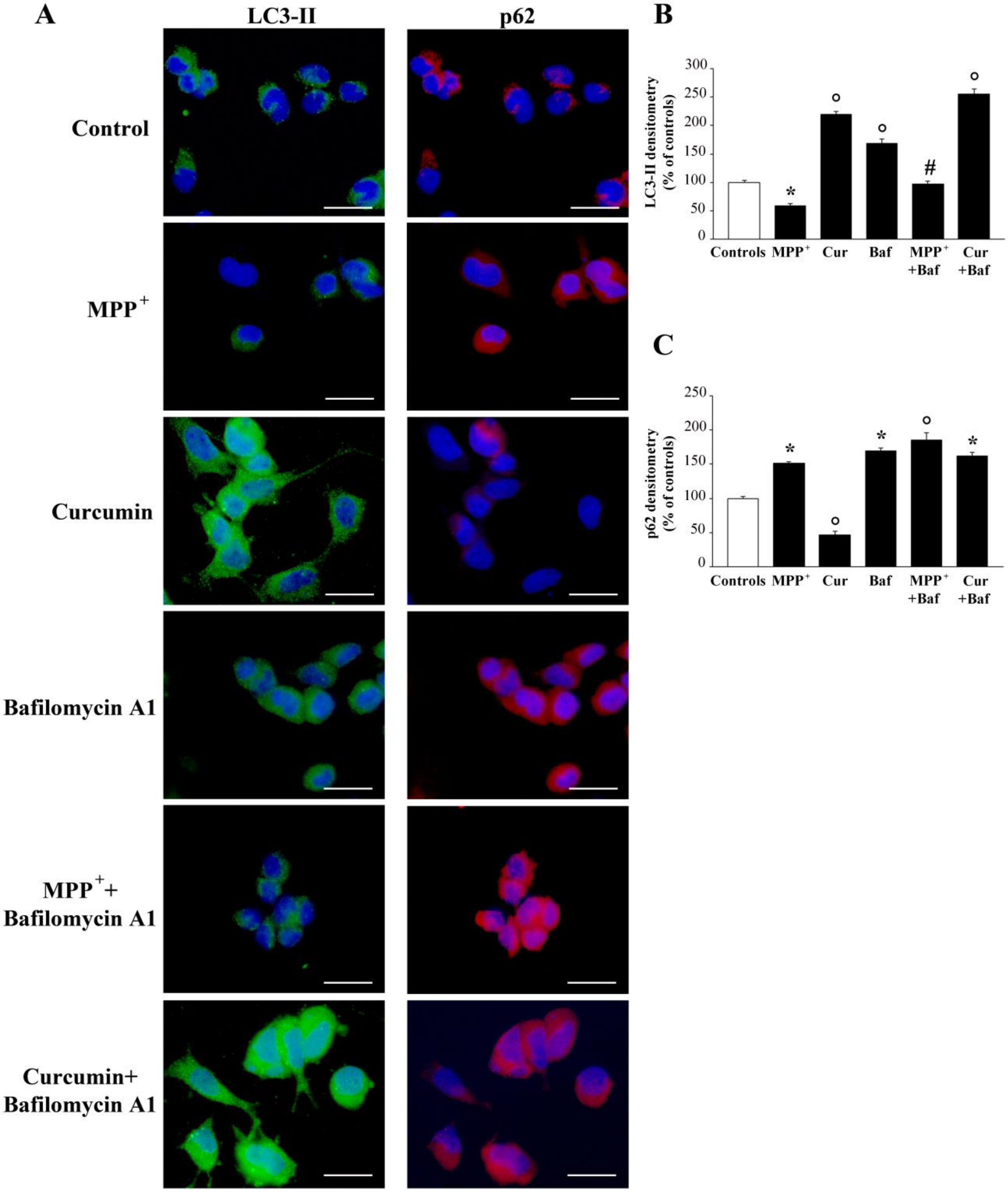


**Supplementary Figure S6.** $MPP^+$ decreases, while curcumin increases, autophagy flux. (**A**) Representative pictures of LC3-II and p62 immunofluorescence from control cells, cells treated with $MPP^+$ 10 μM, curcumin 10 μM, Bafilomycin A1 100 nM or combined $MPP^+$/curcumin and Bafilomycin A1 are shown. Note that

immunofluorescence for LC3-II and p62 was carried out within the very same cells. In $MPP^+$ treated cells, the weak LC3-II immunofluorescence intensity combined with the strong p62 immunofluorescence intensity, both of which are increased by adding the autophagy inhibitor Bafilomycin A1, indicate that autophagy flux is markedly decreased by $MPP^+$, although the persistence of a residual low autophagy flux. In contrast, the high LC3-II and low p62 immunofluorescence intensities observed in curcumin-treated cells, both of which are further increased after treatment with Bafilomycin A1, indicate that curcumin markedly enhances autophagy flux. These findings are supported by the graphs reporting the optical density measurements of LC3-II (**B**) and p62 (**C**). Baf=Bafilomycin A1; Cur=curcumin. *P<0.05 compared with controls; #P<0.05 compared with $MPP^+$; °P<0.05 compared with controls and $MPP^+$. Scale bars=29 μm.

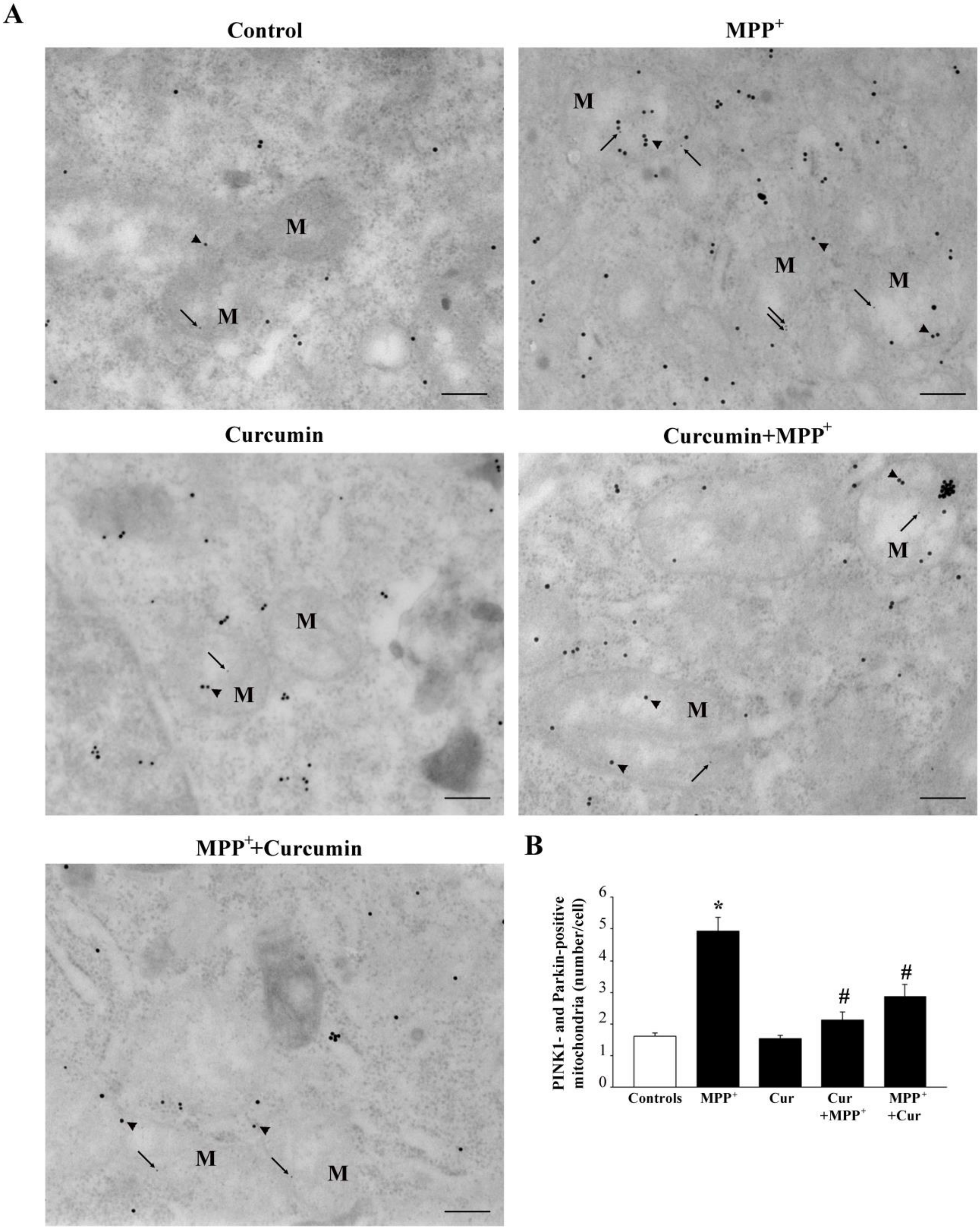


**Supplementary Figure S7.** MPP+ increases, while curcumin decreases, PINK1- and Parkin-positive mitochondria. (**A**) Representative micrographs show from control cells and cells treated with $MPP^+$, curcumin

and combined treatment with curcumin and $MPP^+$, where curcumin is administered before (Cur+$MPP^+$) or after ($MPP^+$+Curcumin) $MPP^+$, show mitochondria labelled with immunogold particles against PINK1 (gold particle diameter 10 nm, arrows) and Parkin (gold particle diameter 20 nm, arrow heads). (**B**) Graph reports the number of mitochondria double labeled with immunogold against PINK1 and Parkin. Cur=curcumin; M=mitochondria. $^*P<0.05$ compared with controls; $^\#P<0.05$ compared with $MPP^+$. Scale bars=269 nm.

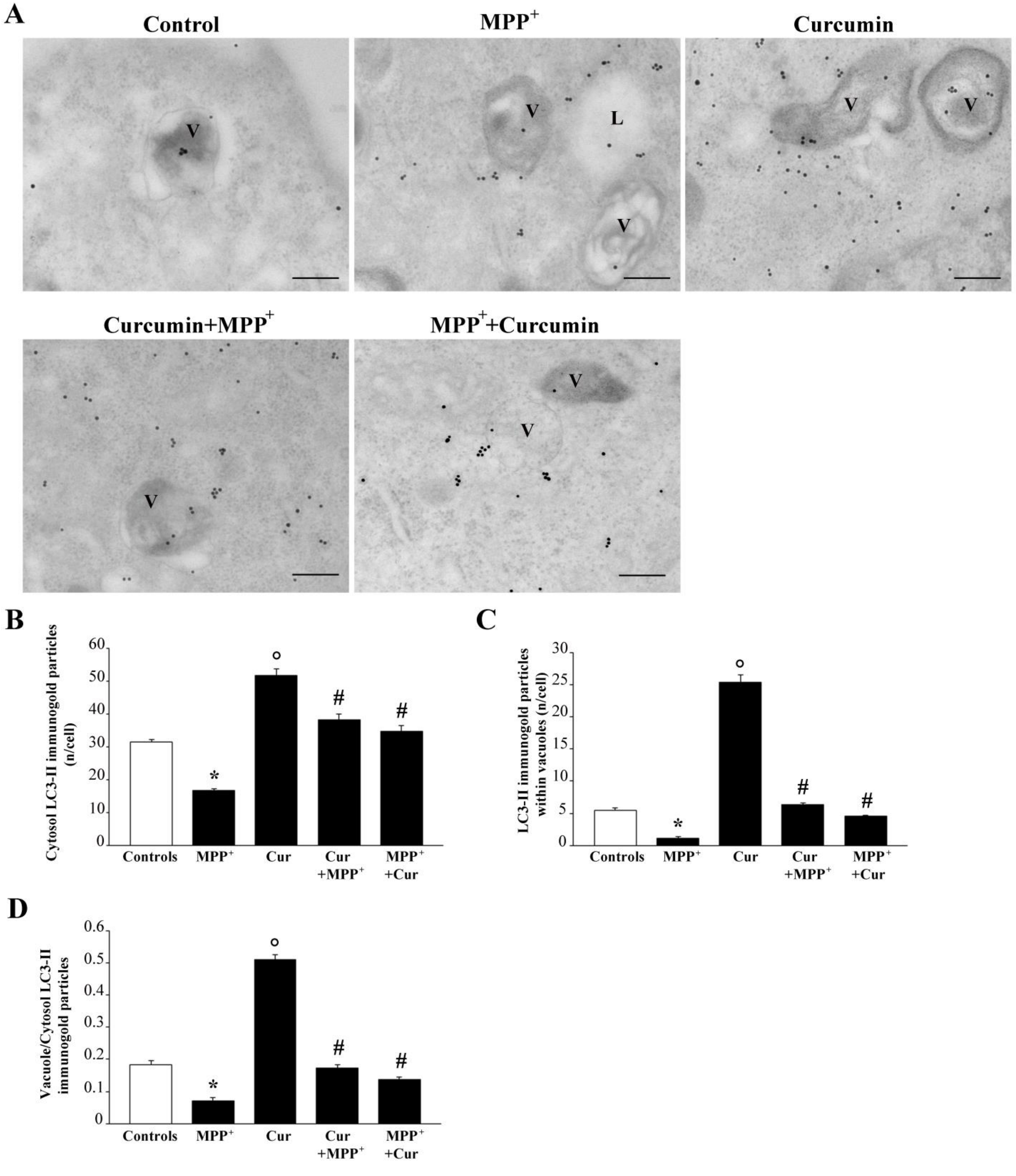


**Supplementary Figure S8.** Opposite effects of MPP⁺ and curcumin on LC3-II intracellular compartmentalization. (**A**) Representative micrographs showing LC3-II immunogold particles within control cells and cells treated with MPP⁺, curcumin and combined treatment with curcumin and MPP⁺, where curcumin

is administered before (Cur+$MPP^+$) or after ($MPP^+$+Curcumin) $MPP^+$. Graphs report: (**B**) the number of LC3-II immunogold particles within the cytosol; (**C**) the number of LC3-II immunogold particles within autophagy-like vacuoles; (**D**) the ratio between LC3-II immunogold particles within autophagy-like vacuoles and LC3-II immunogold particles within cytosol. Cur=curcumin; L=lipid droplet; V=autophagy-like vacuole. *$P<0.05$ compared with controls; $^{\#}P<0.05$ compared with $MPP^+$; °$P<0.05$ compared with controls and $MPP^+$. Scale bars=269 nm.
